# Drug Delivery Systems, CNS Protection, and the Blood Brain Barrier

**DOI:** 10.1155/2014/869269

**Published:** 2014-07-20

**Authors:** Ravi Kant Upadhyay

**Affiliations:** Department of Zoology, DDU Gorakhpur University, Gorakhpur 273009, India

## Abstract

Present review highlights various drug delivery systems used for delivery of pharmaceutical agents mainly antibiotics, antineoplastic agents, neuropeptides, and other therapeutic substances through the endothelial capillaries (BBB) for CNS therapeutics. In addition, the use of ultrasound in delivery of therapeutic agents/biomolecules such as proline rich peptides, prodrugs, radiopharmaceuticals, proteins, immunoglobulins, and chimeric peptides to the target sites in deep tissue locations inside tumor sites of brain has been explained. In addition, therapeutic applications of various types of nanoparticles such as chitosan based nanomers, dendrimers, carbon nanotubes, niosomes, beta cyclodextrin carriers, cholesterol mediated cationic solid lipid nanoparticles, colloidal drug carriers, liposomes, and micelles have been discussed with their recent advancements. Emphasis has been given on the need of physiological and therapeutic optimization of existing drug delivery methods and their carriers to deliver therapeutic amount of drug into the brain for treatment of various neurological diseases and disorders. Further, strong recommendations are being made to develop nanosized drug carriers/vehicles and noninvasive therapeutic alternatives of conventional methods for better therapeutics of CNS related diseases. Hence, there is an urgent need to design nontoxic biocompatible drugs and develop noninvasive delivery methods to check posttreatment clinical fatalities in neuropatients which occur due to existing highly toxic invasive drugs and treatment methods.

## 1. Introduction

The brain is a highly sensitive and fragile neuronal organ system that needs a regular supply of fuels, gases, and nutrients to maintain homeostasis and other vital functions. But BBB a vasculature of the central nervous system acts as a physical barrier and imposes various obstacles. It inhibits delivery of therapeutic agents to the CNS [1] and imposes obstruction for delivery of large number of drugs, including antibiotics, antineoplastic agents, and neuropeptides, to pass through the endothelial capillaries to brain. Though several drug delivery methods and strategies have been developed for CNS related disease therapeutics, most of them are proved invasive and lack the target specificity. More exceptionally, all traditional drug delivery methods are based on trials and errors. These are applied invariably for delivery of few selected drugs that had appropriate structure-activity relationships or drug-receptor interactions, and its structure-transport relationships are intact [2]. However, maintaining normal body functions and transport of various biological substances including therapeutic agents across biological membranes is highly essential [3]. Only few of the existing methods allow drugs for suitable and successful membrane permeation. Moreover, new drug delivery methods are developed based on rational drug design and using high throughput screening receptor-ligand interactions to find appropriateness of the drug among thousands of new compounds. Further, to reduce the postdelivery toxicity of the drugs noninvasive and less toxic drugs and delivery methods have been developed. Hence, a drug should not be selected only after finding high binding affinity to the receptor, in throughput screening, but it must be found suitable on the basis of structure-activity relationships, target receptor binding, and its behavior in animal system. Though it is possible that it may show invariably poor membrane permeation properties* in vivo*, such drugs will undergo insignificant transport through the brain capillary endothelium, which makes up the blood brain barrier (BBB)* in vivo* [4].

There are so many factors, which influence the drug delivery or its ability to traverse the blood brain barrier. Hence, it is possible that drug may bind to nontransporters in larger amount which render the drug ineffective. Second it seems theoretically/falsely active but really it might show the inability to pass through the blood brain barrier with the adhered protein. Therefore, such drugs cannot be made available to the brain because they cannot be transported and delivered across the blood brain barrier. Further, enzyme action also makes the drug inactive or converts it in a nontherapeutic intermediate compound. However, due to solubility reasons membrane barriers disallow larger molecules while smaller molecules are carried over to the brain. Similarly, charged molecules rapidly get into the brain [5]. Therefore, lipophilicity does not seem to be necessary or lonely factor that may assist the drug for safe passage to brain. However, there seems to be a role of multiple factors or complex molecular properties that make drug able to pass through the BBB. More exceptionally, barrier permeability is also related to membrane or luminal surface of brain capillary, composition of CSF or ISF, functional groups, and change on molecular and ionic surfaces, or presence of charged residues of the molecules [6]. In addition, surface activity of the molecules and its relative size and specific binding of transporter proteins and energy driven cassettes and opening and closing of ion channels due to ionic concentration are key factors which play an important role in drug delivery [7].

BBB is nonselective to pass drugs by diffusion or by active transport and creates major hurdles for successful CNS drug development. But it is true that molecules like glucose and fat/lipid soluble drugs can rapidly cross into the brain. Contrary to this, delivery of many of the drug types is very difficult to carry them into the brain because of fat insoluble nature. Besides poor membrane permeation properties, insignificant transport occurs through the brain capillary endothelium affecting the drug availability in theoretically relevant concentration [8]. Major reasons of therapeutic failures are slower drug action, lesser absorption in neuronal and other brain cells, conversion of drug molecule into noninteracting metabolite, and association of drug molecule to other ligands mainly proteins which are nontransporting in nature. Though drug remains therapeutically available in biological system, it becomes ineffective or attains some active molecular form or convert in to a highly reactive molecular state in the brain. This is the main reason why when drug passes through the barrier it might adhere to the unwanted protein in larger amounts [9]. Further, problem may be created by presence of some catabolic enzymes that occur in the brain tissues, which could change the native form of the drug or cleave it into an inactive molecule. There is a possibility that an active drug may change into a slow acting drug molecule that may destructed once it gets inside the brain tissue or enzyme catalytic activity rendering it useless. Therefore, active penetration, structure-activity protection, availability, dispersion, and action of drug in target area are highly needed for the treatment of various CNS disorders and diseases. Further, drug-neuronal receptor interactions, structure-activity relationships, and structure-transport relationships; that is, membrane permeation, must be evaluated for delivery of any drug into the brain.

However, several approaches for direct drug delivery or direct convection-enhanced delivery are used to inject the drug into brain or cerebrospinal fluid or intranasal delivery. These techniques are highly unsafe, invasive local, and metabolizable or short lasting. Contrary to this, there are safe methods which deliver the drug through vascular route which infuse and spread in larger portion of the brain. Hence, for therapeutic purposes active transfer of drug is highly needed. For this purpose safer disruption of BBB or its loosening is highly important to deliver the drug into the brain [10]. Therefore, for successful delivery of drugs, blood brain barrier disruption or opening is done by ultrasound and largely used as intra-arterial infusion therapy. It allows both the chemotherapeutic agents and antibodies to enter through blood brain barrier [11]. Hence, BBB dysfunction could be of great therapeutic value in conditions in which neuronal damage is secondary or exacerbated by BBB damage. However, for therapeutic purposes BBB can be forcibly broken down or disrupted by ultrasonic sound waves for safe delivery of drugs or any therapeutic agent to CNS. But this forced opening may lay structural damage to the BBB and allows the uncontrolled passage of drugs [12]. Further, it is well known that in several areas of the brain BBB is very thin or supposed to be loose or weak, from where drug can easily pass to the brain. These areas also allow passage of important metabolic substances more freely into the brain. These are identified in Pineal body, neurohypophysis, and area postrema. Therefore, by reducing, halting, or reversing the structure and function of BBB new methods can be developed for delivery of chemotherapeutic agents in case of brain tumor. However, in all circumstances both drug composition and its delivery methods [13] must be accounted for making effective drug formulations to treat the CNS disease [8].

**Figure 1:**
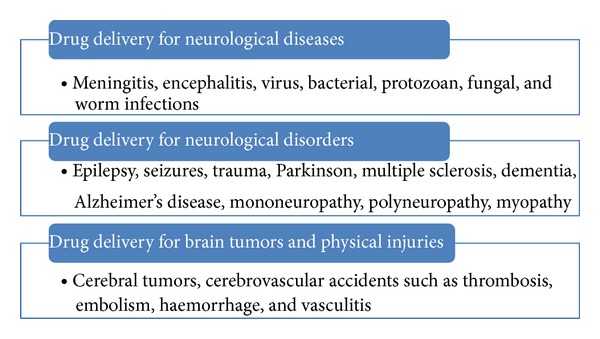
Showing important neurological problems which essentially need proper drug delivery for treatment.

**Figure 2:**
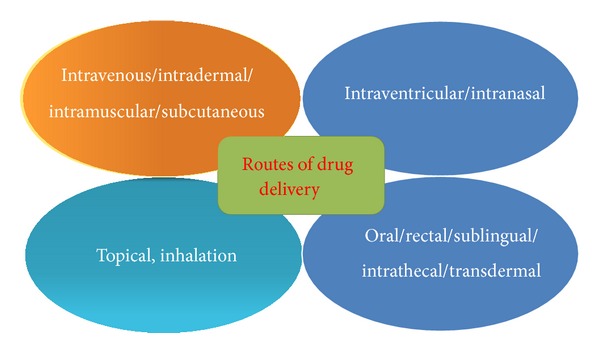
Showing important routes of drug delivery for CNS therapeutics.

So far many different drug delivery methods have been developed. Few of them are delivered neurologically invasive and found unsafe for drug delivery. These are neurological direct injections or structural disruption of BBB by using ultrasound. Other methods which show broad spectrum and deliver wide range of drugs to CNS are pharmacological and physiological methods which are quite safe and noninvasive (Figure 1, Table 1). More specifically neurosurgical strategies include BBB disruption by osmotic imbalance or by using vasoactive compounds, intraventricular drug infusion, and intracerebral implants. In pharmacological methods lipid carrier or liposomes are used for drug delivery. Physiological strategies are followed by applying endogenous transport mechanisms by using either carrier mediated transport of nutrients or receptor mediated transport of peptides. From clinical investigations physiological strategies are proved better and potential delivery methods, because of wider safety cover provided by drug transport. Further, conventional strategies should be improved for safe delivery of different drugs to CNS (Figure 2). These include liposomes, colloidal drug carriers, micelles, chimeric peptide technology, intranasal and olfactory route of administration, and nanotechnology. More specifically, nanoenabled delivery systems offer a promising solution to improve the uptake and targeted delivery of the drugs into the brain.

**Figure 3:**
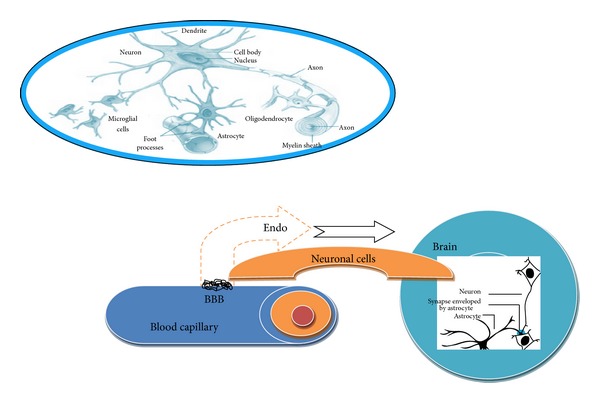
Showing presence of blood brain barrier at the blood capillary endothelium that obstructs drug delivery to CNS.

After delivery of therapeutic biomaterials/pharmaceuticals in the brain its physiological accumulation is needed that plays a crucial role in the treatment of pathogenesis related to neuronal diseases [14]. Another important factor in drug delivery is lipid solubility of drug molecules/compounds that may move across the blood brain barrier by simple diffusion. There are few compounds which could increase the permeability of BBB by loosening the tight junctions between the endothelial cells [15]. Most psychoactive drugs increase the BBB permeability and decrease the physical restrictiveness of endothelial tight junctions and allow most of the therapeutic molecules to pass through the BBB in large amounts (Figure 3). But these drugs are highly invasive and should give only in controlled environment because of the risk of multiple effects. Moreover, over flooding of molecules in brain causes osmotic imbalances and largely affects membrane permeability and blocks or restricts normal supply of nutrients. Second, once tight junctions are loosened, the homeostasis of the brain gets thrown off which results in seizures and imposes compromised brain functions [15]. However, to treat the CNS diseases such as brain tumours, transport protein, peptides, radiopharmaceuticals and other macromolecules are allowed to pass across the blood brain barrier in a controlled concentration. For this purpose nanoparticle delivery methods are proved to be more promising than any other method available. These are most usable and noninvasive methods and proved to be much better than any other conventional method used for the treatment of neurological diseases [16]. Therefore, less toxic bioreversible derivatives of prodrugs, neurohealers, and pharmacological agents are urgently needed. These might enable the safe delivery of variety of drugs including anticancer, antineurodegenerative, and antiviral drugs. More specifically, more sophisticated nanoparticle based tools are required for the treatment of brain tumors, viral and neurodegenerative diseases, and disorders. Present review article aims to emphasize various applications of noninvasive drug delivery methods with recent developments which occurred in nanotherapeutics for CNS protection. Hence, special emphasis has been given to develop nontoxic delivery vehicles and highly soluble, permeable biocompatible anticancer drugs [17] and liposomal carriers to reduce the toxic effects and posttreatment fatalities in case of cancer and brain tumors [17, 18]. In addition, cellular mechanism of drug delivery such as receptor mediated endocytosis, microbubble enhanced focused ultrasound, proline rich peptides, chitosan based nanoparticles, beta-cyclodextrin carriers, cholesterol mediated cationic solid lipid nanoparticles delivery system, Si RA delivery system, colloidal drug carriers, liposomes, and micelles have been discussed with their recent advancements. In addition, suggestions have been given for designing much safer nontoxic delivery vehicles and biocompatible drugs to overcome the problem of clinical failures and posttreatment fatalities [19].

## 2. Cancer and Tumor Therapy

Similar to blood brain barrier, brain tumor microvessels/capillaries also limit drug delivery to tumors by forming a physical barrier [20]. No doubt that TBB is found more permeable than the blood brain barrier [20, 21] but it significantly restricts the delivery of anticancer drugs and obstructs systematic chemotherapeutics of brain tumors [22]. This causes failure of drug target and makes the process extremely difficult to treat solid tumors in the brain. It is the main reason of clinical failures of many effective and potential antitumor drugs. It is usually not due to the lack of drug potency but rather the nondelivery of drug to the brain and into the tumors [23]. Contrary to this, there are few pharmaceuticals which are used in tumor-specific therapies that were found insufficient to check aberrant signaling pathways in brain tumors [24]. It makes the chemotherapeutic treatment ineffective and required amount of drug could not reach into the brain after its delivery [25]. Hence, it is highly suggestive that highly toxic antitumor chemotherapeutic drugs should not be administered in sufficient concentration by conventional delivery methods because these methods were not proved to be much helpful to ascertain long term survival of the patients with brain tumors and most of clinical cases of brain tumors are proving fatal [25]. However, new well-designed safer therapeutic strategies that could deliver an appropriate therapeutic concentration of antitumor drug are to be prepared. These should be more responsive for delivering by applying safer drug delivery systems or methods by breaching any physical and physiological obstacle that exists in the brain [26].

However, for making an easy and successful drug delivery to save the life of tumor/cancer patients many potential techniques were developed [23]. These approaches are intravenous chemotherapy, intra-arterial drug delivery, local drug delivery via implanted polymers or catheters, BBB disruption, and biochemical modulation of drug [26]. Few other drug delivery methods like intracerebroventricular, convection-enhanced delivery are also proved to be highly useful. Further, to enhance the BTB permeability accelerated therapeutic molecules are allowed to pass through it by cellular vasomodulator-mediated transportation mechanism. Thus permeability modulation is possible without BBB/BTB disruption [27]. Interestingly, K(Ca) channels were found to be potential targets for biochemical modulation of BTB permeability that increases antineoplastic drug delivery selectively to brain tumors [22]. Similarly, BTB targeting specific proteins is also used to increase antineoplastic drug delivery to brain tumors [27]. It accelerates with the formation of pinocytic vesicles which assist in transportation of drugs across the BTB. It is also accelerated by using channel activators [21]. Similarly, infused minoxidil sulphate (MS) a selective K(ATP) channel activator comes across the BTB to brain tumor and facilitates delivery of certain macromolecules mainly Her-2 antibody adenoviral-green florescent protein and carboplatin to brain tumors [22]. It has significantly increased the survival in brain tumor rats. Therefore, rat brain tumor models are designed to test enhanced drug delivery to brain following intracarotid infusion of bradykinin (Bk), nitric oxide (NO) donors or agonists of soluble guanylate cyclase (SGC), and calcium dependent potassium K(Ca) channels [21]. Thus modulation of these channels by specific agonists and agents that produce NO and cGMP* in situ* is essentially required. Moreover, selective opening of blood tumor barrier by a nitric oxide donor increases survival in rats [28] and affects cerebral blood flow in intracerebral C6 gliomas [29]. Contrary to this, water soluble compounds are limited by the surface area/permeability of the tumor capillaries [30]. Therefore, in new methods, BBB manipulations are being performed for safe delivery of drug to the brain. These methods are noninvasive which are used in targeted molecular based therapies. Further, multifunctional magnetic nanoparticles magnetic resonance imaging was found to be a highly successful method in cancer therapy [31].

## 3. Use of Prodrugs

Due to presence of physical obstacles imposed by BBB only small amount of drug passes through barrier and reaches to the brain. However, lack of suitable transporter protein slows down the supply of drug into the brain. Therefore, to make the normal drugs medically active, lipophilic molecules are added which make the drug able to pass through the barrier. Thus drug is released in its original and active form into the brain. However, inactive drugs could activate after addition of lipophilic molecules. Further, enzymes due to catalytic action remove the lipophilic group to release the drug into its active form. More often, drugs that cannot pass through the blood brain barrier can deliver into the brain without disrupting the structural barrier by making prodrugs. These are largely used to treat neuronal diseases [32]. Thus, prodrugs can enhance the therapeutic efficacy of drugs and/or reduce adverse effects via different mechanisms, including increased solubility, improved permeability and bioavailability, prolonged half-life, and tissue-targeted delivery [33]. Hence, various prodrug systems, such as lipophilic carriers and receptor mediated prodrug delivery systems, and gene-directed enzyme prodrug systems are used to deliver drugs into the brain [34]. Further, prodrugs, which have no or poor biological activity, are chemically modified to have a pharmacologically active agent, which must undergo transformation* in vivo* to release the active drug [35]. Thus, active prodrug may be able to pass through the barrier and then also repass through the barrier without ever releasing the drug in its active form.

Prodrugs are bioreversible derivatives of drug molecules that undergo an enzymatic and/or chemical transformation* in vivo* to release the active parent drug. These are pharmacologically active agents that overcome barriers to a drug's usefulness. After delivery to the target site prodrugs exert desired pharmacological effect [36]. More specifically inactive drugs or therapeutic compounds are made active by addition of lipophilic groups. These active forms of drug better sneak through the blood brain barrier. These are designed by using most common functional groups that may allow the drug permeability through the physical or any structural barrier device [36]. Prodrugs are used in cancer therapies, including antibody-directed enzyme prodrug therapy (ADEPT) and gene-directed enzyme prodrug therapy (GDEPT) [35]. Other major applications of the prodrug strategy are the ability to improve oral absorption and aqueous solubility, increase in lipophilicity and active transport, and achieve site-selective delivery [35]. These most favoring parameters are essentially required in drug discovery and drug development [36]. In present time about 7–10% of drugs are prodrugs; these are provedto be an effective tool for improving physicochemical, biopharmaceutical, or pharmacokinetic properties of pharmacologically active agents. Further, improvements in basic prodrug design could be made by functional group considerations to drug metabolism involving cytochrome P450 enzymes. It will increase water solubility, bioavailability, permeability, and stability to tumor targeting. It will also assist in the development of new anti-inflammatory anti-HIV agents. Thus by using transporters and receptor mediated endocytosis genes, enzymes and activated prodrugs could be delivered to cancer cells and metastatic tissues [37].

## 4. Peptide Masking

Further, major obstacle to targeting the brain with therapeutics in general (P/P drugs amongst them) is the presence of various barriers. As it is known that blood brain barrier (BBB) controls the concentration and entry of solutes into the CNS. However, for successful permeability P/P drug lipophilicity is required that could be achieved by addition of cholesteryl group that makes them able to pass through BBB. These could be delivered by following intraventricular administration or any other noninvasive method. However, for safe carriage of pharmaceuticals another useful way is masking the drugs by converting its chemical composition into a lipid soluble drug. However, by combining with other molecular groups peptide's basic characteristics are masked and addition of a lipophilic group makes it likely to pass through the blood brain barrier. Hence a cholesteryl molecule is used instead of cholesterol because of its lipophilic nature. It serves to conceal the water soluble characteristics of the drug and such type of masking assists the drug in traversing the blood brain barrier. Similar masking of drug peptide from peptide degrading enzymes also occurs in the brain [32]. However, shorter peptides with good surface charge may bind to the receptors on one side and mask the no passage of larger molecules. However, a target molecule could be attached to the drug; that can easily pass the drug through the BBB. It can increase the drug uptake by the brain. Further, it may degrade in such a way that the drug cannot pass back through the brain. Thus, for complete prohibition of drug reverse transport, it should be converted into a nontransport form and must concentrate in the brain for better therapeutic action [32]. In addition, the drug must be enzymatically degradable that could prevent the overdose to the brain tissue or its removal could minimize the overaction of drug on nervous tissue. Hence, both dosage effect and drug action require intense monitoring [32]. Similarly, C-terminal peptide thioesters also assist in peptide masking. These also affect aminolysis of peptides by the secondary amines used for removal of the Fmoc group. However, backbone amide linker (BAL) strategy is followed for their synthesis in which the thioester functionality is masked as a trithioortho ester throughout the synthesis [38]. It would enhance the effectiveness and delivery of drug. This double-masking of albuterol add-on therapy is used for patients with multiple sclerosis. Similarly, treatment with glatiramer acetate plus albuterol is found to be well tolerated and improves clinical outcomes in patients with multiple sclerosis. But cholesterol masks membrane glycosphingolipid tumor-associated antigens to reduce their immunodetection in human cancer biopsies [39]. Contrary to this, unmasking by permeabilizing but nondetaching treatment with cholesterol-binding detergents digitonin and edelfosine compares with and overlaps that of PAO phenylarsine oxide [40]. However, depletion of the surface sites by N-terminally clipped Y2 agonists indicates larger accessibility for a short highly helical peptide. It shows the presence of a dynamic masked pool including majority of the cell surface Y2 receptors in adherent CHO cells [40]. However, in spite of their potential, many existing peptide and protein drugs (P/P drugs) are rendered ineffective in the treatment because of their inability to deliver and sustainability within the brain. For high accessibility, masking molecules should be of low molecular weight of 400–500 Da so that they can easily cross the BBB and deliver the drug in pharmacologically significant amounts [32, 41, 42].

## 5. CNS Protection

### 5.1. Intranasal Delivery of Drugs

There are so many drugs that reach the CNS after nasal administration in different animal models as well as in humans [43] (Figure 2). However, to deliver sizable amount of drug into the brain intranasal administration of neuroprotective agents is found to be more useful for the treatment of ischemic brain injury. It is a preferable method used to deliver local ailments of cold cough, rhinitis, and so forth [44]. Further, to accelerate the action of drug colloidal nanoparticles mucosal or tumor barrier intranasal delivery method is applied to send them to various parts of brain. But delivery of peptides and proteins seems to be very hard to send them for systemic use through nasal route [44]. Moreover, for delivery of peptide and proteins various more appropriate nanoparticles are required [44]. When a nasal drug formulation is delivered deep and high enough into the nasal cavity, it reaches to olfactory mucosa and transport into the brain and/or CSF via the olfactory receptor neurons. It should generate good immune response due to preferential interaction to the lymphoid tissue of the nasal cavity (NALT). However, drug transport through olfactory epithelium [45] should work as a conduit for transmission of drugs to the CNS but, drug transfer in animals show substantially different ratios of olfactory-to-respiratory epithelium than humans [46]. Moreover, two possible routes, that is, the olfactory nerve pathway (axonal transport) and the olfactory epithelial pathway [47], are followed by the drugs to reach into the brain. Moreover, soon after nasal delivery of a drug it first reaches to the respiratory epithelium, where it absorbed into the systemic circulation by trans-cellular and para cellular passive absorption, or by transcytosis or endocytosis [47, 48]. However, absorption across the respiratory epithelium is the major transport pathway for nasally administered drugs. It may represent a potentially time saving route for the administration of certain systemic drugs delivered in cryonics medication protocols (e.g., epinephrine or vasopressin). But sometimes BBB-mediated exclusion of brain-therapeutic agents also remains unsuccessful and drug is found to be diffused in unwanted regions. Hence, to overcome this problem carbopol-based gels are made for nasal delivery of biopharmaceuticals [49].

However, intranasal administration of NAD+ is found to be neuroprotective as it decreases transient focal ischemia [50]. Similarly, intranasal administration of the PARG inhibitor gallotannin also decreases ischemic brain injury in rats [51]. Such agents abolish activation of poly(ADP-ribose) polymerase-1 (PARP-1), which plays a significant role in ischemic brain damage. Further, NAD+ was observed to reduce infarct formation by up to 86% even when administered at 2 hours after ischemic onset [51]. Similarly, intranasal administration of antiporters or NMDA receptor blockers provides neuroprotection against the more upstream events of global ischemia such as membrane depolarization and excitotoxicity [52]. Similarly, nasal administration of EPO (erythropoietin) is a potential, novel, neurotherapeutic approach in the treatment of acute ischemic stroke in humans [53]. It is one of the most successful methods that show neuroprotective capacity in the treatment of patients with acute stroke and other neurodegenerative disorders. No doubt that this new therapeutic approach could revolutionize the treatment of neurodegenerative disorders in the 21st century [53].

Moreover, brain possesses two drug passing routes for transportation of substances; one is axonal transport that ranges from 20–400 mm/day to a slower 0.1–4 mm/day [54]. It is considered to be a slow route whereby an agent enters the olfactory neuron via endocytotic or pinocytotic mechanisms and travels to the olfactory bulb by utilizing the same anterograde axonal transport mechanisms. Cell uses transport endogenous substances to the brain by this mechanism [47]. The epithelial pathway is a significantly faster route for direct nose-to-brain transfer, whereby compounds pass paracellularly across the olfactory epithelium into the perineural space, which is continuous with the subarachnoid space and in direct contact with the CSF. Then the molecules can diffuse into the brain tissue or will be cleared by the CSF flow into the lymphatic vessels and subsequently into the systemic circulation [45, 55]. Similarly, nasal spray method could increase the quantity of VIP (vasoactive intestinal peptide) entering the brain and protect the central nervous system. Hence, drugs sent through intranasal route cause minor irritation, which resolve spontaneously within a week at the end of the treatment [56]. More often, intranasal delivery is a noninvasive, safe (Figure 2, Table 1), and alternative approach which rapidly targets delivery of molecules to the brain while minimizing systemic exposure [57].

### 5.2. Intraventricular Drug Delivery

Intraventricular drug delivery is used for pain medication and drug is delivered within the cerebrospinal fluid of the cistern (C1-2 vertebra) and intracranial ventricles. This method is primarily used for delivery of analgesic drugs for patients having, tumors of head, face, and neck. More often it is used in cerebral drug targeting [63] by administering medication directly. It needs less amount of drug and imposes fewer side effects than orally administered drugs. In this methods a plastic reservoir is used, which is implanted subcutaneously in the scalp and connected to the ventricles within the brain by an outlet catheter. Thus, medicine is delivered through this implanted catheter connected to a pump that may be programmable and either implanted or external. For example, insulin is directly targeted into the brain via intracerebroventricular (ICV) or intraparenchymal delivery (Figure 2). It is an invasive technique with significant risk, necessitating repeated surgical intervention and providing potential for systemic hypoglycemia [57]. This method aids in clinical therapeutics of associated neurodegenerative and neurovascular disorders (Figure 1) [57].

Similarly, intraventricular delivery of rituximab activates complements C3 and C5b-9 in CSF. It shows an improved efficacy of intraventricular immunotherapy both via modulation of the innate immune response and innovations in drug delivery [64]. Similarly, intraventricular injections of folate receptor-*α*-positive and -negative exosomes into mouse brains demonstrate folate receptor-*α*-dependent delivery of exosomes into the brain parenchyma [57]. Furthermore, vascular endothelial growth factor promotes pericyte coverage of brain capillaries that improve cerebral blood flow during subsequent focal cerebral ischemia and preserves the metabolic penumbra [65]. It also enhances cerebral blood flow during subsequent ischemic episodes, leading to the stabilization of cerebral energy state. It is possible that it induces the formation of new vessels and improves brain tissue survival [66]. Similarly, hypothalamic neuron-derived neurotrophic factor acts as a novel factor which modulates appetite, food intake, body weight, increased hypothalamic Pomc, and Mc4r mRNA expression [67]. Importantly, the appetite-suppressing effect of NENF was abrogated in obese mice fed a high-fat diet, demonstrating a diet-dependent modulation of NENF function [68]. Similarly, antiangiogenic pigment epithelium-derived factor (PEDF) a multifunctional 50 kD secreted glycoprotein promotes stemness by upregulation. Moreover, intraventricular injection of PEDF promotes stem cell renewal, while injection of VEGF initiates differentiation and neurogenesis in the subventricular zone [69]. Hence, enhancing the expression of PEDF in stem cells has promising therapeutic implications because this protein possesses several bioactivities in nearly all normal organ systems. It will be an essential component in the development and delivery of novel stem cell-based therapies to combat disease [68].

Similarly, intraventricular delivery of vancomycin is used to treat meningitis, ventriculitis, and CNS associated infections. However, disposition of vancomycin within CNS aids in the improvement of pathophysiological conditions, strokes, and injuries that will facilitate in better understanding of the effects on pharmacokinetic and pharmacodynamic parameters of neuroactive drugs in adults [68]. Further, it is proved by fluorescence microscopy studies that FITC-D3 accumulates in the vacuolar compartments of the cells and can be detected in various structures and populations of cells after injection into the brain. Similarly, convection-enhanced delivery into the putamen nucleus [70], PDA, pressure support, surfactant therapy, inotropic drug administration, vaginal delivery, neonatal resuscitation, and antenatal corticosteroid therapy could be more significantly used higher in cases with IVH (intraventricular hemorrhage) [71]. It is mainly used to treat hyaline membrane disease and preeclampsia in mother [60]. Similarly, intravenous, intracerebroventricular, or intranasal administration of siRNA to neurons, glia, and brain capillary endothelial cells (BCECs) is used to treat neurological diseases [72]. Gene silencing therapies are also used to deliver short interfering RNA (siRNA) into central nervous system (CNS) while polylysine dendrimers D3 and D5 [73] and melittin-grafted HPMA-oligolysine based copolymers are also used for gene delivery [73]. Similarly, melittin-containing polyplexes are also found to be promising biomaterials for gene delivery to the brain [73]. Moreover, Gd-DTPA diffusion in gliomas could assist in real-time monitoring of interstitial drug delivery and quantitative assessment of biophysical structural variations in diseased tissue [73]. Further, G4 PAMAM dendrimer distribution patterns in the CNS may facilitate the design of tailored nanomaterials in light of future clinical applications. It does not induce apoptotic cell death of neural cells in the submicromolar range of concentration and induces low microglia activation in brain tissue after a week [74].

### 5.3. Use of Peptide Radiopharmaceuticals

Radiolabeled receptor-binding peptides and proteins have emerged as an important class of radiopharmaceuticals that have changed radionuclide imaging in clinical practice. These have increased the diagnostic potential of neuroimaging technology and are proved to be a more sophisticated diagnostic tool to scan brain for Alzheimer's disease. More importantly, in brain imaging small-molecule radio chemicals that bind to monoamine or amino acid neurotransmitter systems are used. For example, epidermal growth factor (EGF) peptide radiopharmaceuticals were found to be potential candidates for neuroimaging which are used for early detection of malignant gliomas or brain tumors [75, 76]. Similarly, PET imaging is also used for detection of neuroendocrine tumors [77] in which heterodimeric molecule is used for primary and recurrent prostate cancer covering. These two receptor entities might lead to an improved diagnostic sensitivity and therapeutic efficiency [78]. Similarly, peptide-based (18)F-radiopharmaceuticals (Table 1) are used for diagnostic applications with positron emission tomography (PET) in clinical trials [73]. In addition, tailored gallium (III) bioconjugation is also widely used in preclinical Ga-68-PET Imaging [79].

However, for neuroimaging many strategies have been developed to radiolabel peptides and proteins with fluorine-18. It is a more straightforward approach based on the chelation of aluminum fluoride by (1,4,7-triazacyclononane-1,4,7-triacetic acid). Thus, use of Al(18)F labeling technology has optimized yield and specific activity and neuroimaging potential of peptides [80]. NOPO-functionalized peptides provide suitable pharmacokinetics* in vivo *[81]. In addition, inverse electron-demand Diels-Alder click chemistry is used to develop novel radiopharmaceuticals [82]. Similarly, chemoselective labeling of the integrin ligand-c(RGDyK) peptide-has been developed on the basis of the Cu(I)-catalyzed conjugation reaction. Moreover, nucleophilic detagging and fluorous solid-phase extraction method provides an easy way to implement an approach for obtaining 2-[(18)F] fluoroethyl azide [83]. Similarly, development of A*β* peptide radiopharmaceutical combined with a nanocarrier works as molecular Trojan horse and has wider applications* in vivo* amyloid imaging in Alzheimer's disease [84]. Similarly, (99 m) Tc-peptide-ZHER2:342 molecular probe is a promising tracer agent used for visual detection of cancer [85]. Similarly, (131)I-tRRL small peptide because it specifically binds to tumor-derived endothelial cells [62]. Moreover, Tc-EDDA/HYNIC-E-[c(RGDfK)]2 obtained from kit formulations showed high tumour uptake in patients with malignant lesions. It is a promising imaging marker that is used for targeting site-specific breast cancer [86]. Moreover, (18)F-glyco-RGD peptides are used in PET imaging of integrin expression, modulation, and biodistribution. Recently integrins have become increasingly attractive targets for molecular imaging of angiogenesis with positron emission tomography or single-photon emission computed tomography, but the reliable production of radiopharmaceuticals remains challenging [87].

It is very difficult to map the functional connectivity of discrete cell types in the intact mammalian brain during behavior. Cell type based designer receptor maps exclusively prepared by seeing their interactions using designer drug (DREADD) technology could clearly differentiate between brain functions in normal and disease states. Hence, behavioral imaging with *μ*PET and [18F] fluorodeoxyglucose (FDG) can generate whole-brain metabolic maps of cell-specific functional circuits during the awake and freely moving state. More often, DREAMM could reveal discrete behavioral manifestations and concurrent engagement of distinct corticolimbic networks associated with dysregulation of Pdyn and Penk in MSNs of the NAcSh. DREAMM is a highly sensitive, molecular, high-resolution quantitative imaging approach that could clear any brain disorder [88]. PET imaging of tumors with a 64Cu labeled macrobicyclic cage amine ligand tethered to Tyr3-octreotate. MeCOSar is a promising bifunctional chelator for Tyr3-octreotate that could be applied to a combined imaging. Thus therapeutic regimen can be prepared by using a combination of (64)Cu- and (67) and CuSarTATE complexes, owing to improved tumour-to-nontarget organ ratios compared to (64)CuDOTATATE at longer time points [89]. PET with 62Cu-ATSM and 62Cu-PTSM is a useful imaging tool for hypoxia and perfusion in pulmonary lesions [58]. Further, amount of (18)F-FDG uptake is determined by the presence of glucose metabolism, hypoxia, and angiogenesis [90, 91].

### 5.4. Use of Protein Neurotherapeutic Agents

BBB restricts entry of many potentially therapeutic agents (PNA) into the brain. But recently, several neuroactive proteins of potential therapeutic value have highlighted the crucial need for effective and safe transcapillary delivery methods to the brain. However, most promising drug delivery is possible by augmentation of pinocytotic vesicles through brain capillaries. This is a cellular mechanism which assists in delivering large molecules of neurotherapeutic potential in conjugated form like peptidomimetic ligands. Later on these molecules bind to selected peptide receptors, which internalize and transport (PNA) in small vesicles across the cytoplasmic brain capillary barrier. These conjugates are found to be functionally active and effective in animal models of neurological disease. In fact all neuroprotective small molecules have failed to repair stroke in clinical trials because either these molecules have unfavorable safety profiles or the drugs do not cross the BBB. When properly delivered, these provide neuroprotection up to 3 hours after stroke, during which BBB remain intact [92]. These short peptides showed favorable safety profiles in brain after coming cross the BBB [93]. For example, neurotrophin, a brain derived neurotrophic factor (BDNF), is reformulated to enable BBB transport. Similarly, BDNF chimeric peptide was found to be neuroprotective following delayed intravenous administration in either regional or global brain ischemia [92–96]. Similarly, erythropoietin a novel neurotherapeutic agent [97] is also a primary physiological regulator of erythropoiesis [97], exerts effect by binding to cell surface receptors, and displays hormonal role. It is produced by the kidney in response to hypoxic stress and signals the bone marrow to increase the number of circulating erythrocytes [98]. In addition, both erythropoietin and its receptor found in the human cerebral cortex, astrocytes, and neurons that are members of a cytokine superfamily mediate diverse functions in nonhematopoietic tissues. It shows neuroprotective activity that is upregulated following hypoxic stimuli. Similarly, in animal models, exogenous recombinant human erythropoietin was proved to be beneficial in treating global and focal cerebral ischemia and reducing nervous system inflammation in experimental animals [99]. Erythropoietin dramatically reduces postinfarct inflammation and shows healing effect in brain and repairs spinal cord injuries such as mechanical trauma, experimental autoimmune encephalitis, or subarachnoid hemorrhage. It directly modulates neuronal excitability and acts as a trophic factor for neurons* in vivo* and* in vitro*. It shows dose-dependent effects and is highly beneficial in epileptic or degenerative neurologic diseases [100], because erythropoietin generates potential impact on biodistribution of drug and shows fast action mechanism when it passes through BBB [100]. Therefore, pharmacological exploitation of erythropoietic agents could provide therapeutic benefits in CNS dysfunction [100]. However, delivery of anthraquinone-2-sulfonic acid (AQ2S) acts as a novel neurotherapeutic agent against cerebral ischemia that protects the brain from strokes and neurological diseases [59, 101].

Besides, neuroprotective compounds monoclonal antibodies are also used as novel neurotherapeutic agents to repair CNS injury caused by trauma or hyperthermia [102]. In such injuries level of serotonin (5-HT), dynorphin A (Dyn A 1–17), nitric oxide synthase (NOS), and tumor necrosis factor-*α* (TNF-*α*) increases, that also acts as potential neurodestructive signals in the CNS injury. Thus, for neutralization of these agents monoclonal antibodies directed against 5-HT, NOS, Dyn A (1–17), and TNF-*α* *in vivo* can be used for neuroprotection and to enhance the neurorepair after trauma [102]. Similarly, activation of the nuclear factor E2-related factor 2/antioxidant response element pathway is neuroprotective after spinal cord injury [103]. Similarly, Epo and the Epo receptor (EpoR) play a critical role in neurodevelopment, neuroregulation, and neuroprotection. It ameliorates and prevents neuronal injury and shows neuroprotective, antiapoptotic, anti-inflammatory, antioxidant, angiogenic, neurogenic, and neurotrophic effects in cell culture and animal models [98].

Similarly, metallothioneins (MTs) is a superfamily of highly conserved, low molecular weight polypeptides, which are characterized by high contents of cysteine (sulphur) and metals. These are intracellular metal-binding proteins which play a significant role in the regulation of essential metals [104]. In both central and peripheral nervous tissues, MT-I and MT-II have neuroprotective roles, which are also induced by exogenous MT-I and/or MT-II treatment. Both MT-I and MT-II may provide neurotherapeutic targets offering protection against neuronal injury and degeneration [104]. In addition, metallo-complexes formed inside brain may possess enough potential for treatment of neurodegenerative diseases [105]. Similarly, testosterone shows neuroprotective effects on morphology in both males and females. It also acts as a neurotherapeutic agent in the injured nervous system [106]. Similar to testosterone androgen also regulates neuritin mRNA levels in an* in vivo* model of steroid-enhanced peripheral nerve regeneration [107]. Similarly, indomethacin-loaded lipid-core nanocapsules reduce the damage triggered by A*β*1–42 in Alzheimer's disease models and this blockage of neuroinflammation triggered by A*β* is involved in the neuroprotective effects of IndOH-LNCs. It is a promising approach for treating AD [108].

### 5.5. Use of Chimeric Peptides

However, transport of therapeutic peptides through BBB remains a challenge for peptide drug delivery into the central nervous system (CNS) (Table 1). However, chimeric peptides carry the drug into the brain to targeted sites though it does not transport through the BBB. For this purpose drug is conjugated to a brain drug-targeting vector [109]. These chimeric proteins easily pass through BBB and presence of these peptide drugs inside cell could be detected by immune-fluorescent markers. Chimeric protein consists of a protein of interest covalently linked to naturally fluorescent proteins that enable biologists to image movements of industrial proteins in living cells. However, by using rDNA technology a chimera of any desired protein of interest linked to a naturally fluorescent protein and express inside a cell or an organism can be prepared.

However, tumor necrosis factor receptor-IgG fusion protein is prepared for targeted drug delivery across the human blood brain barrier. The tumor necrosis factor-alpha receptor (TNFR) contains an extracellular domain (ECD) that can be used in neurotherapeutics of stroke, brain injury, or chronic neurodegeneration [101, 110]. As nascent TNFR ECD is a large therapeutic molecule that does not cross the blood brain barrier (BBB), it was reengineered by fusion of the receptor protein to the carboxyl terminus of the chimeric monoclonal antibody (mAb) to the human insulin receptor (HIR). This fusion makes it able to decoy receptor transportable across the human BBB [110]. Similarly, metabolically stable opioid peptide [3H]DALDA ([3H]Tyr-DArg-Phe-Lys-NH_2_) was also prepared that is used as a model drug which transports through the BBB into brain extracellular fluid [111]. However, cleavable disulfide linkers are used in the synthesis of such “chimeric peptides.” It is crucial to save S-S-bridge intact and stable during transcytosis. However, cleavage within endothelial cells could result in sequestration of the drug moiety instead of passage through the BBB [111]. It was monobiotinylated with the cleavable biotin reagent sulfosuccinimidyl 2-(biotinamido) ethyl-1, 3′-dithiopropionate (NHS-SS-biotin) to obtain bio-[3H]DALDA. The biotinylated peptide is then bound to a vector for brain delivery after intravenous injection in rats, a covalent conjugate of streptavidin, and the transferrin receptor monoclonal antibody, OX26. Moreover, the most common strategy which is followed to treat moderate to severe pain consists of the activation of opioid receptors in the brain. Hence, the development of active opioid peptide analogues as potential analgesics requires compounds with a high resistance to enzymatic degradation and an ability to cross the BBB.

Moreover, monoclonal antibody-glial-derived neurotrophic factor, a fusion protein, penetrates the blood brain barrier in the mouse. Similarly, majority of the fusion proteins are transcytosed across the BBB with penetration into brain parenchyma. It was confirmed by brain capillary depletion analysis [112]. Similarly, tetrapeptide analogues of the type H-Dmt1-Xxx2-Yyy3-Gly4-NH_2_ are transported into the brain after intravenous and subcutaneous administration and are able to activate the *μ*- and *δ* opioid receptors more efficiently and over longer periods of time than morphine [113]. Similarly, therapeutic elevations of GDNF could also be achieved in mouse brain with intravenous administration of the cTfRMAb-GDNF fusion protein [112]. Moreover, a brain penetrating IgG-erythropoietin fusion protein was constructed which shows neuroprotective effects following an intravenous treatment in Parkinson's disease in the mouse [114]. Parkinson's disease (PD) is caused by oxidative stress, and erythropoietin (EPO) reduces oxidative stress in the brain. However, to make EPO cross the blood brain barrier (BBB) a brain penetrating form of human EPO has been developed. EPO is fused to a chimeric monoclonal antibody (MAb) against the mouse transferrin receptor (TfR), which is designated as the cTfRMAb-EPO fusion protein. The TfRMAb acts as a molecular Trojan horse to transport the fused EPO into brain via transport on the BBB TfR [114]. Similarly, avidin (AV) is fused to the carboxyl terminus of the heavy chain of the genetically engineered chimeric monoclonal antibody (mAb) against the mouse transferrin receptor (TfR). The TfRMAb binds the endogenous TfR on the blood brain barrier (BBB), which triggers transport into brain from blood. This cTfRMAb-AV fusion protein is a new drug delivery system that can target to mouse brain monobiotinylated peptide or antisense radiopharmaceuticals [114]. More specifically IgG-avidin fusion protein assists in delivery of a peptide radiopharmaceutical to brain [114].

Thus, both recombinant fusion peptides and proteins are used as drugs which have shown great therapeutic efficacy against various neurodegenerative diseases. But transport of these molecules (P/P drugs) through blood brain barrier (BBB) is still a major challenge because of their larger size [115]. Contrary to this smaller drugs have not been effective neuroprotective agents in either the acute treatment of stroke such as focal brain ischemia or the chronic treatment of neurodegeneration even after their larger permeability across BBB [93]. More often, large molecule drugs such as recombinant neurotrophins, and neurotrophins do not cross the brain capillary endothelial wall but prove to be more effective than smaller size drugs. Hence, to make neurotrophins transportable across the BBB, chimeric peptides are made in which a neurotrophin is reformulated by fusion to a transport vector. Transport vector is a peptide or peptidomimetic monoclonal antibody that undergoes receptor mediated transcytosis through the BBB and acts as a molecular Trojan horse [93]. Similarly, glial-derived neurotrophic factor (GDNF) is a neurotrophin that could be developed as a agent for treatment of Parkinson's disease, stroke, and motor neuron disease [61]. Therefore, by reengineering of GDNF, neurotrophin was made transportable across the human BBB by fusion of the mature GDNF protein to the carboxyl terminus of the chimeric monoclonal antibody (MAb) to the human insulin receptor (HIR) [61]. However, peptides or protein therapeutics may be delivered to the brain with the use of the chimeric peptide strategy. However, to make chimeric peptide strategy successful, vector development and coupling of drugs to the vector and liberation of biologically active peptides following cleavage of the bond linking are important steps [116]. Furthermore, avidin/biotin system is proved to be more advantageous in fulfilling these criteria for successful linker strategies. However, OX26 monoclonal antibody are used in avidin/biotin system and a vasoactive intestinal peptide (VIP) analogue is fused to make it suitable for monobiotinylation and retention of biologic activity following cleavage [116]. In addition, in chimeric peptide delivery method proteins such as cationized albumin or the OX26 monoclonal antibody are used as transport vectors and bound to the transferrin receptor. These proteins undergo absorptive-mediated and receptor mediated transcytosis through the BBB, respectively (Table 1) [116].

Moreover, endogenous peptide, modified protein, or peptidomimetic monoclonal antibody (mab) that undergoes RMT (Rapid metabolic transfer) through the BBB on endogenous receptor systems such as the insulin receptor or the TfR is also used. Interestingly, this peptidomimetic mabs bind to exofacial epitopes on the BBB receptor that is removed from the endogenous ligand binding site and piggyback across the BBB. Drug is monobiotinylated and fused with a vector/avidin or a vector/streptavidin (SA) fusion protein [109]. Because of extremely high affinity of avidin or SA binding of biotin, there is instantaneous capture of the biotinylated neurotherapeutic agent made by the vector/avid in or vector/SA fusion protein [117]. Furthermore, monoclonal antibody/avidin and mab/SA fusion genes and fusion proteins are produced by using genetic engineering methods that are proved to be good delivery methods in humans [118].

### 5.6. Disruption of BBB by Using Focused Ultrasound

For fast action of a drug its successful delivery in to the brain and its proper distribution is highly essential. Furthermore, for safe and noninvasive distribution of drug reversibly at targeted locations needs disruption of blood brain barrier (BBB). This BBB disruption is induced by pulsed ultrasound in the presence of preformed gas bubbles. It is operated very carefully because over pitch sound may harm brain tissues. Therefore, sonication should be provided in a controlled manner to make it noninvasive and reversible to deliver the drug at targeted locations without inducing substantial vascular damage (Table 1). Because ultrasonic results in ischemic or apoptotic death to neurons [119], it has emerged as an important diagnostic technology that is used for localized and reversible disruption of the BBB for treatment purposes [1]. It has wider applications in molecular neurooncology [24]. Similarly, ultrasound induced MRI guided BBB disruption could also be possible for drug delivery into the brain [1]. Similarly, few other strategies are also in developing phase like burst ultrasound which is performed in the presence of an ultrasound contrast agent that also disrupts BBB by using acoustic waves in the selected region of the brain. HRP injected in the brain passes through MRI induced BBB disruption at pressure amplitude between 0.4 MPa and 1.4 MPa [120]. Further, EM that demonstrated HRP passage through vessel walls via both transendothelial and paraendothelial routes proves disruption. It is a much safer method for targeted drug delivery than any other convection method employed for drug delivery [120, 121]. Both of these techniques have emerged as noninvasive methods. No doubt that diagnostic technology based on MR (magnetic resonance) imaging assists in monitoring of therapeutic agents, their distribution, and kinetics in neuronal tissues (Table 1) [122].

Some other strategies such as radiation therapy or chemotherapy are used for tumor therapeutics, which do not provide good prognosis tumor progression control or improved patient survival [122]. Further, temporal disruption of the BBB by microbubble-enhanced focused ultrasound (FUS) exposure can increase CNS blood permeability providing a promising new direction to increase the concentration of therapeutic agents in the brain to control tumor formation, necrosis, and tissue invasiveness. It shows no long term adverse effect and provides longevity in the patients. Further, for BBB break-down mannitol solution is injected into arteries in the neck that results in high uptake of sugar by brain capillaries, which also takes up water out of the endothelial cells, shrinks them, and opens tight junction. This effect lasts for 20–30 minute, during such time drugs diffuse freely, that would not normally cross the BBB. This method permitted the delivery of chemotherapeutic agents in patients with cerebral lymphoma, malignant glioma, and disseminated CNS germ cell tumors [117, 123]. In addition, disruption or damage of endothelium could allow expression of endothelial receptors which are normally downregulated, opening new communication loops between endothelium, pericytes, astrocytes, and microglia. These also play an important role in barrier repair. Physiological stress, transient increase in intracranial pressure, and unwanted delivery of anticancer agents to normal brain tissues are the undesired side effects observed in man.

### 5.7. Loaded Microbubble Enhanced Focused Ultrasound

Besides the above methods, blood brain barrier can be temporarily and locally opened by focused ultrasound in the presence of circulating microbubbles [124]. Microbubbles are small “bubbles” of monolipids that are able to pass through the blood brain barrier. They form a lipophilic bubble that can easily move through the barrier [119]. The ultrasound increases the permeability of the blood brain barrier by causing interference in the tight junctions in localized areas. Thus combined effect of microbubbles and ultrasonic sound allows drug into a very specific area with the diffusion of microbubbles. More often, microbubbles diffuse only where the ultrasound disrupts the barrier. Focused ultrasound is also used to deliver targeted NK-92 cells to the brain using a model of metastatic breasts cancer [125]. Thus loading a microbubble with an active drug to diffuse through the barrier and target a specific area increases the usefulness and action of drug [119]. It was also found to be more feasible for targeted gene transfer into central nervous system by MRI guided focused ultrasound induced blood brain barrier disruption [126]. Similarly, doxorubicin-loaded microbubble technology has been developed that destroys tumors with focused ultrasound and makes fragments. Further nanoshards formed are capable of escaping through the leaking tumor vasculature, promoting accumulation of drug within the interstitium [127]. Thus hydrophilic drug doxorubicin and paclitaxel loaded microbubbles are used for ultrasound triggered drug delivery [127]. Similarly, hydrophobic drug paclitaxel loaded UCA (polymer ultrasound agents) triggered with focused ultrasound showed enormous potential for targeted and sustained delivery of drug to tumors [127]. Instead of microbubble size, its route and stability must be determined for delivering the drugs to specific sites in the brain (Table 1) [119].

Similarly, for safer and efficient drug delivery NPs (nanoparticles) are used as one of the major potential delivery vehicles to carry drug and distribute it in various locations in human body via different pathways. Therefore, strategies, which could successfully transfer nanoparticle to brain, may significantly improve the efficacy of neuroprotective drugs in brain stroke [128] and neurodegenerative disease [129]. These could also be used to release oxidative stress generated after pathogenesis [130], though brain contains high oxygen metabolism but lacks an antioxidation protection mechanism [130]. However, oxidative stress associated with gene expression analysis can provide efficient information for understanding neuroinflammation and neurodegeneration associated with NPS [130]. Thus, dysfunction of blood brain barrier (BBB) will assist in drug delivery and carry it to major targets of pathological sites [131]. It also enhances drug concentration and its therapeutic action assists in treatment of CNS related diseases, disabilities, and disorders which seem to be very difficult to treat [129]. Further, both receptor and site of action of drug at BBB require better drug designs that could not only enhance its activity and selectivity but also make significant increase in the therapeutic index of drug [129] (Table 1).

Further, the size of the drug molecule seems to be a major determinant factor in CNS therapeutics. Whether a substance absorbs and comes across the nasal respiratory epithelium and/or transports along the olfactory pathway it needs a perfect smaller size [132]. Other factors which affect the drug delivery to the brain include the degree of dissociations and lipophilicity. However, higher lipophilicity may result in better transportation of therapeutic agents. Once a drug is transferred in the brain, it is further influenced by BBB efflux transporter systems like P-glycoprotein (P-gp) [133]. Its uptake into the brain could be enhanced when drugs are administered in combination with the P-gp efflux inhibitor, rifampicin [48, 134]. Further, there is no effective therapeutic intervention developed to check cerebrovascular toxicity of drugs of abuse such as methamphetamine [135]. Similarly, to enhance antioxidant capacity of cerebral microvessels intensive physical exercise could protect against METH induced disruption of blood brain barrier [135]. However, phospholipid enclosed vesicles released by both eukaryotes and prokaryotes into their environment remove harmful molecules by vesicle cargos. These could be used to exchange biomolecules by loading on transmembrane receptors. These also deliver genetic information by same route and same mechanism [136]. These vesicles protect cell from accumulation of wastes and drugs inside the cell. Microvesicles have many chemical applications and are used as biomarkers in cancer therapy [136]. These vesicles easily pass through blood brain barrier and act like naturally occurring liposomes and endowed drugs may transfer to brain and persist for a longer period. Thus drug persistence for longer duration protects brain from virus infection, injuries [136], cancer, and certain epilepsies [137]. Moreover, equilibrium must be established between cerebrovascular permeability when a drug is transferred via the circulatory system for the therapy of neurodegenerative diseases. However, to avoid different barrier inhibiting CNS penetration by the therapeutic substances various drug delivery methods such as chemical drug delivery and carrier mediated drug delivery have been established [129].

Furthermore, contrast enhanced microbubble ultrasound is a noninvasive method which is used for assessment of breast lesions [138]. These are detected prior to larger bubbles following decompression [139]. Gas microbubbles are highly comprehensive, but phospholipid coated microbubbles generate large change in resonance frequency. These are used for measurement of small blood pressure variations in deep blood vessels [139] and absolute blood pressure in surface organs [139]. However, lipid shelled microbubbles and albumin shelled microbubbles are used to deliver drug to breast cancer cells [140]. Similarly, biotinylated microbubbles [141] and methylene microbubbles are used in dual modality ultrasound and activatable photoacoustic imaging [142] and in sonothrombolysis [143]. Therefore, ultrasound stimulated drug delivery is done for treatment of residual disease [144]. Similarly, drug perfusion enhancement in tissues could be achieved by steady streaming induced by oscillating microbubbles [145]. Further, enhanced delivery of micro-RNA mimics cardiomyocytes using ultrasound responsive microbubbles resurfaces hypertrophy in an* in vitro* model [146]. However, combination of bubble liposomes and high intensity focused ultrasound and microbubble guided drug delivery [147] are used for tumor ablation [147, 148]. Thus, use of ultrasound induced disruption and microbubbles could successfully transfer nanoparticle to brain; that may significantly improve neuroprotective efficacy of drugs in brain stroke [129] and neurodegenerative disease [130].

## 6. Drug Delivery Methods

### 6.1. Proline Rich Peptides as Delivery Vehicles

Certain proline rich peptides which pass through blood brain barrier are used for treatment of cerebral infections [149]. Best example is oncocin that after entering into brain 80% of it is trapped in the endothelial cells while other peptides such as drosocin and apidaecin Api 137 reached into the parenchyma cells and were found stable in the plasma and brain [149]. Bryostatin a potent protein kinase c (PKC) activator showed brain therapeutic efficacy [150]. Similarly, dolichyl-P increases transendothelial transfer of Rhodamine 123 (Rh 123) and Ab 42 from the apical compartment to the basolateral compartment [14]. Thus, its accumulation in the brain exerts an important role in the depression of p-gp at the BBB and promotes function of the pump at the BBB in AD. Similarly, anthocyanins found in berry fruits are active phytochemicals which show reversion of age related cognitive impairment and protect against neurodegenerative disorders [151]. Hence, this is more plausible that mechanism of neuroprotective action of anthocyanin may be via modulation of signal transduction processes and/or gene expression in the brain tissue [151]. Similarly, CFC-C showed significant neuroprotective effect as it contained various components on apoptosis related proteins. However, flavonoid and polysaccharide components in Jiawei Wuzi Yanzong formula can pass through the blood brain barrier and protect neurons from beta amyloid protein induced neurons up to some extent [138].

Similar neuronal protective efficacy is also observed in* Astragali radix* (AR) by oral administration against Japanese encephalitis virus (JEV) infection in mice. However, in AR treated mice peritoneal exudates cell (PEV) or macrophage numbers get increased and active oxygen production was obtained high [152]. It shows a significant increase in survival rates in animal groups with RA and this effect was found to be dependent on a nonspecific mechanism during the early phase of infection [152]. Similarly, Quin Wen oral liquid protects the experimental rabbits facing hemorrhagic fever [153]. It delays the incubation period, lowering down febrile index and PGE context. It improves hemorheology and enhances the cell mediated immunity in CSF [153]. Similarly, arginase 1 has been shown to protect motor neurons from trophic factor deprivation. It allows sensory neurons to overcome neurite outgrowth inhibition by myelin proteins. Similarly, daidzein consumed with soya products crosses the blood brain barrier and appears to be safe and effective without any pretreatment. It can be developed as an ideal candidate for development of therapeutic drugs for spinal cord injury or strike. Similarly, glutamate antagonists were found to be highly useful and are used to protect neural tissues against Ischemia. The antagonists such as magnesium, MK 801, and combination of magnesium and MK 801 reduce brain edema and restore BBB permeability after experimental diffuse injury [154]. Similarly, oximes are used to mitigate O. induced neuronal injury. They restart or reactivate inhibited organophosphate local AChE [155]. Similarly, subfragments of amyloids beta appear to protect neurons from Alzheimer's disease [156]. Moreover, Chitosan microspheres are used to trap the drug and form a nanocarrier for its permeation through the BBB. It is a novel method mostly used in nanovaccine delivery [157]. It can be used to deliver drugs to treat virus infection dementia [158] and neurocognitive disorders (Table 1) [159]. This is also used to activate angiotensin converting enzyme (AE) inhibitors those which cross blood brain barrier [159]. Similarly, erythropoietin (EPO) also acts as a neuroprotector that is used through intranasal delivery [45, 157]. It is a noninvasive method which bypasses the blood brain barrier (BBB) in order to deliver therapeutic agents to brain [157]. More specifically, N acetylcysteine amide (NACA) protects the blood brain barrier (BBB) from oxidative stress inducing damage in gp 120 Tat and methamphetamine treated animals [160]. Thus, it could become viable therapeutic option for patients with HIV-1 associated dementia (HAD) [160]. In addition antiretroviral treatment prevents central nervous system dysfunction by decreasing brain viral load and interferon alpha levels [159].

### 6.2. Nanoparticles as Drug Delivery Vehicles

**Figure 4:**
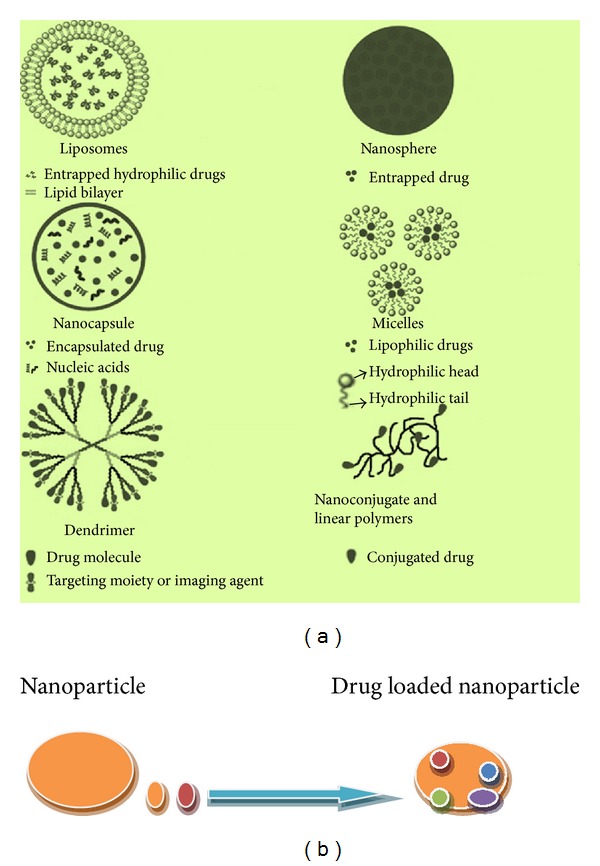
(a) Showing structures of different types of drug delivery vehicles, (b) a drug loaded nanoparticle.

Nanoparticles are nanoscale sized polymeric particles which are made up of natural or artificial polymers. These are ranging in size between about 10 and 1000 nm (1 mm). These interact with biological barriers and easily pass through it and are used for drug targeting and biodistribution of pharmaceuticals in a controlled manner. Drugs can bound in form of a solid solution or dispersion or adsorbed to the surface or chemically attached on nanoparticles support carrier loading (Figure 4). Further, polymer used in construction of nanoparticles improves their stability in the biological environment. It also assist to mediate the biodistribution of active compounds, drug loading, drug targeting, transport, release, and interaction with biological barriers. But in normal cases use of nanopolymers is proved to be invasive and toxic as their degradation products create serious problems in the CNS. However, cytotoxicity generated by nanoparticles or their degradation products remain a major problem in drug development. However, valid improvements in biocompatibility are much needed; hence it should be the main concern of future pharmaceutical research [161].

Nanoparticles have enormous medical applications and emerged as the major tools in nanomedicine than conventional drug delivery methods [162]. These provide massive advantages regarding drug targeting, delivery, and release. Further, their additional potential can be harnessed to combine diagnosis and therapy, which will work as much usable emerging tools in nanomedicine [163]. These are proved to be best delivery vehicles to carry drugs to biological systems for a safer therapeutics of variety of neurodegenerative and virus generated diseases. These are highly efficient drug delivery systems that are potentially used for many applications, mainly in antitumors therapy, gene therapy, AIDS therapy, and radiotherapy. These are also used for delivery of proteins, antibiotics, virostatics, and vaccines and are used as carriers or vesicles to pass the blood brain barrier [162, 163]. In addition, these drug delivery systems have potential use in transfer of molecular and immunological agents to the biological system. These are used for gene delivery and to make recombinant therapeutic peptides synthesized by fusion of new genes into the cells. It can ably transfer neurotrophic agents to abolish neurodegenerative diseases. Thus, nanoparticle permeation allows safe and sustained release of drug at the targeted site after 1 or 2 weeks of injection [164]. More specifically nanoparticles have wider application in brain tumor therapy and treatment of cancer and Alzheimer's disease [165].

There are two main categories of nanoparticles, inorganic and organic. These are mentioned in Table 2. Inorganic nanoparticles are mainly magnetic, metallic, nanoshells, and ceramic. Magnetic nanoparticles are super paramagnetic iron oxide particles that display large magnetic moments in a magnetic field. These are biocompetitive, noncompatible chemically stable, and easy to manufacture. These are mostly used for targeted delivery of drugs/genes and are used in thermotherapy. Next category of nanoparticles is metallic nanoparticle which comprises gold or silver or copper and iron nanoparticles. These are smaller in size (<50 nm) having large surface area, carry high drug doses, but these show poor biocompatibility and have no decided function when used* in vivo*. These are used for controlled release of drugs, proteins, and DNA encapsulated in hollow cores of metal shells at desired sites. These are widely used in catalysis, sensing, imaging, and drug delivery. Silica nanoparticles are nanoshells that possess similar imaging/therapeutic potential as quantam. These are less toxic and are relatively large in size compared with quantam dots. These are used for photothermal tumor ablation. These form immunoconjugates which are highly applicable for immunoglobulin bioassay. Ceramic nanoparticles are made up of nonmetallic materials that are cheap and stable. These can be formed by inorganic biocompatible materials, silica, titania, and alumina. These are of smaller size (<100). These are relatively flexible, easy to manufacture, water soluble, and biologically stable. These can form coatings and make bulk materials at low temperatures.

**Figure 5:**
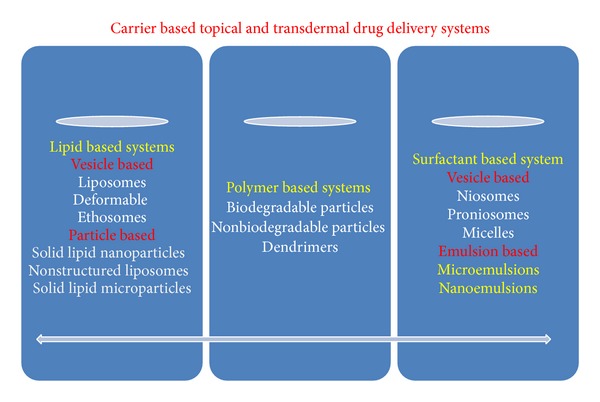
Showing topical and transdermal drug delivery systems.

**Figure 6:**
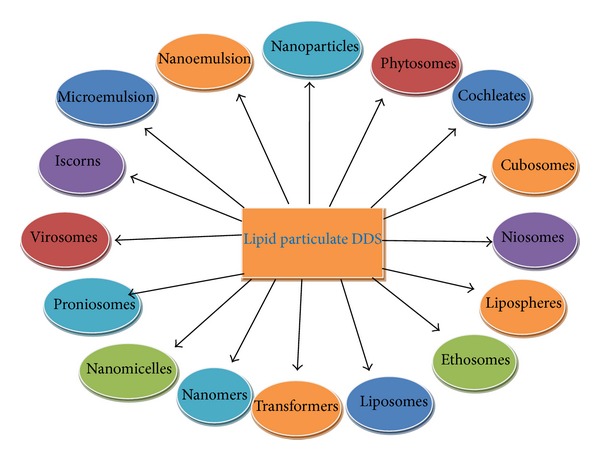
Showing different types of liposomes used for drug delivery to CNS.

Many types of organic nanoparticles such as carbon nanotubes, quantam dots (semiconductors), dendrimers, liposomes, and polymeric nanoparticles have been made (Table 3). These are crystalline form of pure carbon. Carbon nanotubes are graphite sheets rolled into single or multiwalled tubes. Carbon nanotubes are used in electromagnetic shielding of polymers composite for hydrogen, storage, and its batteries. These are used for targeted delivery of drugs, genes, and vaccines and are widely used in thermotherapy of tumors. Quantam dots are semiconductor crystals formed by combination of chemical elements from groups II, III, and V of the periodic table. These are made up of cadmium core and metal shell and have similar size <10 nm. These are used* in vitro* labeling of live cells and for gene expression studies, fluorescent imaging assays to detect antigens or cells. These are used for* in vivo* cancer diagnosis. Dendrimers are highly branched macromolecules synthesized through polymerization reactions. These are used for targeted delivery of genes, proteins, and peptides. Liposomes are closed spherical assemblies of amphipathic phospholipid bilayer. These are nontoxic, biodegradable, and nonantigenic in nature. These are used for controlled release of drugs packed within liposomes or intercalated into lipid bilayers. Polymeric nanoparticles are colloidal nanoparticles which are made up of biodegradable polymer matrices. These are used for delivery of plasmid DNA, proteins, peptides, and low molecular weight compounds. These are mostly used to deliver water insoluble drugs (Table 3). Lipid-based, polymer based, and surfactant based carrier systems have been developed for topical and transdermal drug delivery (Figure 5). Other modifications of liposomes such as PEGylated liposomes, niosomes, and aquasomes are also used for targeted drug delivery (Figure 6).

However, different nanoscale carrier systems have been made by using number of materials such as poly(alkylcyanoacrylates) (pacas), polyacetates, polysaccharides, and copolymers for an easy and efficient drug delivery. Four different types of nanoparticles are constructed; these are coated nanoparticles, PEGylated nanoparticles, solid lipid nanoparticles, and nanogels. Mostly, polyalkyl poly(alkylcyanoacrylates), polyacetate, polysaccharides and copolymers are used in construction of nanoparticles and for making efficient drug delivery system. Nanoparticles made of biodegradable polymers such as polylactic acid, polycaprolactone, poly(lactic-co-glycolic acid), the poly(fumaric-co-sebacic) anhydride chitosan, and modified chitosan, as well as solid lipids, have shown great potential in the delivery of proteins/peptidal drugs. However, poly(butyl cyanoacrylate) nanoparticles are used for* in vivo* drug delivery to the brain successfully. In some cases it is reported to mimic molecules that would normally be transported to brain. For example, polysorbate-coated nanoparticles are thought to mimic low-density lipoprotein (LDL), allowing them to be transported across the capillary wall and into the brain by loading on the LDL receptor [166, 167]. Further, size and construction material not only increased their efficacy but also improved the action of drug or any other pharmaceutical agent across the barrier [162, 163, 167]. It allows sustained drug release at the targeted site after injection over a period of days or even weeks [164]. In addition, new hydrogels and transdermal drug delivery systems are to be developed for peptidal drug delivery [168]. The first drug that was delivered to the brain using nanoparticles was the hexapeptide dalargin (Tyr-D-Ala-Gly-Phe-Leu-Arg), a Leu-enkephalin analogue with opioid activity.

Nanoparticle based delivery methods are proved to be the best methods to transfer drugs across CNS [12]. These strategies require multifunction NPs combining controlled passage across the BBB. These are proved to be the best methods to facilitate the delivery of drugs and biological therapeutics for brain tumors across the BBB [12]. Nanoparticles could easily traverse the BBB and carry drug to targeted locations inside brain and tumor. A better example is HAS (human serum albumin), that is used as nanoparticle. It is well tolerated to the patients and shows no serious side effect. More exceptionally albumin functional groups can be utilized for surface modification of barrier that allows specific cell uptake [165]. It also acts like as a transforming growth factor in microbubble based drug delivery [166]. Further, to enhance the effectiveness of nanoparticles, these are coated with certain biodegradable materials which make them more permeable to cross the blood brain barrier. However, lipid shelled and nonlipid shelled nanoparticles are prepared [169–171]. Similarly, biodegradable polymeric nanoparticles [172], transferrin-conjugated, fluorescein-loaded magnetic nanoparticles [173], solid lipid nanoparticles [169], and chitosan based nanoparticles [174] were made for targeted delivery of drugs across the blood brain barrier. Similarly, hydrogel-based ionotropic delivery devices are also developed for transdermal delivery of peptide/protein drugs [175]. Still it is a challenging task for nanotechnology in delivery of imaging preface in biological systems [176]. However to improve the drug release and its biodistribution and for enhancing the therapeutic applications and efficacy ester prodrugs are incorporated into the nanoparticles [171]. These are also coated with different hydrophilic or hydrophobic drug materials [177]. Mostly, polysorbate-coated nanoparticles are used to deliver drug to the brain as these showed better efficacy than uncoated nanoparticle [177]. Furthermore, nanolipid carriers and solid lipid nanoparticles are used as colloidal drug carriers for different therapeutics [178].

Because of their smaller size nanoparticles penetrate into even small capillaries and are taken up within cells. Thus, after delivery, an efficient drug accumulation takes place at targeted sites in the body [167]. However, to enhance the therapeutic action of drug its maximum absorption in the tissues and organs is required. Though, exact mechanism of nanoparticle transport into brain is not understood, it is thought to depend on the particles size, material composition, structure, and design of nanoparticles. In some cases it is reported to mimic molecules that would normally be transported to brain. Further, for targeting cancerous brain tumors Photofrin is used along with iron oxide into nanoparticles. Photofrin is a type of photodynamic therapy (PDT), in which the drug is drawn through the blood stream to tumors cells. Further, a special type of laser light activates the drug to attack the tumor. Iron oxide is a contrast agent that is used to enhance magnetic resonance imaging (MRI). Therefore nanoparticle based strategies have been developed to establish equilibrium between cerebrovascular permeability outside and inside of nerve cells.

### 6.3. Chitosan Based Nanoparticles

Chitosan based nanoparticles (NPs) require suitable drug carrier which could deliver the pharmaceuticals to the various parts of neurocompartments [179]. Interestingly, chitosan NPs easily enter neuronal cells by endocytosis and transfer through membrane bound vesicles and free in the cytosol and accumulate around the nucleus [179]. However, for sustained surge of certain hormones chitosan-nanoconjugated hormone nanoparticles [180] such as insulin nanoparticles are prepared for oral delivery [181]. Similarly, Smrho protein loaded chitosan nanoparticles [182] and chitosan-sodium lauryl sulfate nanoparticles [183] are also prepared for oral delivery of insulin and other therapeutic agents [182, 184]. In addition, chitosan-Pluronic nanoparticles are used as oral delivery of anticancer gemcitabine [185]. Similarly, low molecular weight chitosan nanoparticulate system at low N : P ratio are also prepared for nontoxic polynucleotide delivery [186]. Further, different types of nanoparticles such as chitosan-DNA nanoparticles [187], lecithin/chitosan nanoparticles [188], chitosan-alginate [189], and chitosan-coated iron oxide nanoparticles are also prepared for sustainable drug delivery [190]. Moreover, 5-aminolevulinic acid-incorporated nanoparticles of methoxy poly(ethylene glycol)-chitosan copolymer are used in photodynamic therapy [191], while, FVIII-chitosan nanoparticles [192], cyclosporin A-loaded, PEGylated chitosan-modified, lipid-based nanoparticles [193, 194], and chitosan and poly(lactic-*co*-glycolic acid) incorporated nanoparticles (heparin) are also prepared for quick CNS therapeutics [195]. Similarly, thiolated chitosan nanoparticles are also prepared for drug delivery system for antisense therapy [196] (Table 2).

Further, for improving the therapeutic and pharmacological efficacy of drugs its natural structure is protected by encapsulation. It makes the drug able to cross biological barriers and carry it to intracellular target sites [179]. Besides this, brain penetration may enable the drugs in controlled state that will minimize the overdose effect and accessibility of drug candidate into the CNS compartment [197]. Further, required accumulation of drug needs appropriate and prospective drug design based on normal delivery principles to save the CNS from xenobiotic substances or its adverse effects [197]. Therefore, in new therapeutics nanoparticles allow sustained release of drug into brain critically needed for treatment of CNS related diseases (Figure 2) [198]. It can ably transfer neurotrophic agents for curing many neurodegenerative diseases of central nervous system (CNS). In addition, for treatment of neurological disorders novel drug candidate should be identified [199] and more approachable drug design with higher drug action and its possible effects in brain tissues are enumerated [197]. In addition, nanoparticle based gene delivery vehicles could transfer genes to restore neurodegenerative disease like Alzheimer's, Parkinson's, and Epilepsy and brain tumors. Further, nanoparticle generated cytotoxicity should be evaluated in animal models like Zebra fish [200].

### 6.4. Beta-Cyclodextrin Carriers

Similarly ammonium beta-cyclodextrin (QA beta CD) nanoparticles are used as drug delivery vehicles/carriers for doxorubicin (Dox), a hydrophobic anticancer drug across the blood brain barrier (BBB) (Figure 5, Table 2) [201]. Bcrp (barrier cancer resistance protein) a major component of the blood brain barrier is located on endothelial cells near the tight junctions [202]. It lacks in Sertoli cells and is known as blood testis barrier (BTB); instead, it is localized to the endothelial tight junction in microvessels in interstitium and peritubular myeloid cells in the tunica propria [202]. Bcrp is an ATP dependent efflux transporter [202]. Similarly, l-arginine in inclusion complexes of omeprazole with cyclodextrins [203] makes a hydrophobic pharmaceutical mediated self-assembly of *β*-cyclodextrin containing hydrophilic copolymers. It is used as nanovehicles for neuroactive drug delivery (Table 2) [204]. Many cyclodextrin based nanoparticles have been prepared which show different physicochemical properties and dissolution. Further, cyclodextrin based nanosponges have been made for delivery of resveratrol [205]. In addition, few important *β*-cyclodextrin inclusion complexes are prepared by using dexamethasone acetate-*β*-cyclodextrin [206], amoxicillin *β*-cyclodextrin, [207], ethyl cellulose-coated amoxicillin/chitosan-cyclodextrin-Based Tablets [208], and piroxicam-*β*-cyclodextrin [209]. Further, improvement in dissolution behavior of poorly water soluble drug was done by using cyclodextrin in extrusion process [210]. Similarly inclusion complex of novel curcumin analogue CDF and *β*-cyclodextrin was prepared to enhance* in vivo* anticancer activity against pancreatic cancer [211]. Similarly, sulfobutyl ether *β*-cyclodextrin (SBE_7_ *β*-CD) carbamazepine complex was prepared that showed* in vivo* antiepileptic activity [212]. Moreover, mechanism of adding/removing acetyl groups to histone lysine residues is one of many epigenetic regulatory processes which control the expression of genes; many of them are essentially required for neuronal survival [213].

### 6.5. ATP Binding Cassettes

The ATP binding cassettes (ABC) transporters are important selective elements of the blood brain barrier (Table 2). These occur over the laminal plasma membrane of the brain capillary endothelium facing the vascular space [214] and protect against toxic effects by limiting drug delivery to the brain [170]. These selectively bind to neurotoxicants and prevent entry of neurotoxicants by limiting their accessibility into brain parenchyma [214]. These operate through multiple signaling pathways following of expression and activity of P-glycoprotein. ABC transporters are modulated in response to xenobiotics, stress, and disease [214]. Further, deficiency of P-glycoprotein at the BBB inhibits the efflux activity of certain biomolecules at the blood brain barrier which also protect the brain from overdose [14]. However, increased transporter expression occurs in response to signals that activate specific transcription factors including pregnane a receptor, constitutive androstane receptor, nuclear factor kappa beta, and activator protein 1 [214].

ABC transporter proteins with the aid of energy derived from ATP hydrolysis are used to export a large variety of drugs from the cytosol to extracellular medium. ABC transporter proteins are expressed in many different cell types from different organs but exceptionally these are expressed in luminal cells and multidrug resistant transport proteins in case of tumor and cancer cells. Further, expression of ATP driven efflux transporters in barriers and excretory tissues is regulated by certain ligand activated nuclear receptors [170]. Similarly, Mrp 2 multidrug resistance associated protein 2 and breast cancer resistance protein (BcRP) and CAR are detected and expressed in rat and mouse brain capillaries [170]. Moreover, CAR activation selectively tightens the blood brain barrier by increasing transporter activity and protein expression of three xenobiotic efflux pumps [170]. Similarly, a constitutive androstane receptor is also identified as positive regulator of p-glycoprotein [206]. The p-glycoprotein (p-gp), multidrug resistance protein, and the breast cancer resistance protein (BCRP) are members of the ATP binding cassette transporter family of proteins that is responsible for rapid transportation of drug across the cell membrane that regulates both uptake and efflux [215]. However, overexpression of these transporters particularly p-gp affects the distribution of drugs in various parts of the body including the central nervous system (CNS). It is also responsible for the development of drug resistance in cancer cells [215].

However, reduced function and expression of gPgP result in slow clearance of neurotoxic peptides such as amyloid beta peptide from the neuronal cells [215]. P-gp is thought to send back circulating toxic compounds from brain to blood circulation. Moreover, drugs recognized by efflux transporters including ATP binding cassette transporter such as p-glycoprotein (MDR1/ABCB1), breast cancer protein (BCRP/ABCG2), and multidrug resistant protein-4 (MRP4/ABCC4) show low permeability across the brain barrier resulting in low distribution to the brain [216]. Thus brain to blood efflux transport system also plays an important role in the clearance of endogenous neurotropic compounds such as prostaglandin and beta amyloid whose reduction is related to disorders of the CNS [216]. Similarly, dolichyl-P in the brain plays an important role in the depression of the P-gp at BBB that results in increased pump function at the BBB [14]. Therefore use of neuroprotective agent, that is, brain derived neurotropic factor (BDNF) which protects neurons against these effects, could be of immense therapeutic importance [217]. Thus development of a drug delivery system that can cross BBB may have significant therapeutic advantage [217]. However, preparation of magnetically guided nanocarrier may provide viable approach for targeting BDNF across BBB. These could transmigrate across the BBB. However, such nanocarriers can be used as potential therapeutic carriers to treat opiate addiction neurotoxic effects and synaptic degeneration in patients [217]. Therefore, few drugs, which maintain blood to brain influx transport systems, for example, an amino acid transporter Lat1/SLC 7A5 and organic cation transporter, show CNS delivery [216]. Thus brain to blood efflux transport systems also play an important role in the cerebral clearance of endogenous neurotoxic compounds such as prostaglandins and beta amyloid [216].

### 6.6. Cholesterol Mediated Cationic Solid Lipid Nanoparticles Delivery System

Lipid-based nanoparticle formulations are used as drug carriers [218] for peptides and proteins [219] and for oral administration of drugs [220, 221]. Lipid-derived nanoparticles are also used for immunostimulatory RNA adjuvant [222] and transdermal drug delivery [223] (Table 3, Figure 5). Similarly, cationic lipid/DNA lipoplexes [224], PLGA-based nanoparticulate systems [225], light-sensitive lipid-based nanoparticles [226], and multifunctional lipid-coated nanoparticle are used for cancer therapy [227] while polylipid nanoparticles [228] and cyclen-based cationic lipids are used for more efficient gene delivery towards tumor cells [229]. Similarly, both functional lipids and lipoplexes are used for improved nonviral vector gene delivery [230, 231] (Figure 5, Table 3).

Similar to lipid nanoparticles mainly cholesterol mediated cationic solid nanoparticles (CSLNS) were formulated with esterquat (EQ1) and stearylamine which act as positively charged external layers on hydrophobic internal cores of cacao butter. Thus an increase in the weight percentage of cholesterol and EQ1 promote the uptake of SQV-CSLNS by HBMECs and high content of cholesterol. Moreover, EQ1 in SQV-CSNLS increased the BBB permeability of SQV [232]. Therefore, cholesterol mediated SQV-CSNLS can be more efficacious drug delivery system for brain targeting delivery of antiviral agents [232]. Layer-by-layer thin film of reduced graphene oxide and gold nanoparticles are used in laser-induced desorption/ionization mass spectrometry for effective detection and drug delivery [233]. Similarly, diketopiperazine-based motif is considered as a novel brain shuttle for the delivery of drugs with limited ability to cross the blood brain barrier [225, 234]. It works as an ideal candidate for the retinoid development of new therapeutic agents. Its derivatives also show remarkable neuroprotective and nootropic activity [234] in experimental animal models [234]. Similarly, activated astrocytes protect neurons from toxic substances and can be used for protection of CNS from various chemotherapeutic agents/drugs. Normally, these are used for treatment of fatal disease [235]. In addition, there is an urgent need of nanovehicles for intracellular delivery systems [236]. Further, stem cell therapy combined with technology could become a promising tool to deliver drugs to brain tumors more efficiently (Table 3).

### 6.7. SiRNA Delivery System

Liposomal siRNA nanocarriers are used for cancer therapy [237, 238] and to suppress effects of oncogenes [239] (Table 3), though, it is a great challenge to use multifunctional nanoparticles delivering small interfering RNA to overcome drug resistance in cancer cells [240]. These liposome-siRNA peptide complexes are prepared by incorporating a small peptide that binds SiRNA and acetylcholine receptors (AchRs) acting as a molecular messenger for delivery to neurons and cationic liposomes that protect SiRNA peptide complexes from serum degradation [241]. Thus, LPSCs (liposome-SiRNA peptide) complexes which deliver PrP SiRNA specifically to Ach-R-expressing cells suppress PrP© expression and eliminate PrP siRNA throughout the brain [241]. LPSc were found to be effective vehicles for delivery of PrP and other SiRNA specifically to neurons to treat neuropathological diseases [241]. Similarly, small RNAs of virus and host origins have been found to modulate virus host interactions by RNA interference (RNAi), leading to antiviral immunity or viral pathogenesis [242]. These distinct classes of small RNAs guide specific gene silencing at both transcriptional and posttranscriptional levels and serve as specificity determinants [242]. Similarly, nucleolin-targeting liposomes guided by aptamer AS1411 are used for the delivery of siRNA for the treatment of malignant melanomas [243]. Anti-VCAM-1 SAINT-O-Somes enable endothelial-specific delivery of SiRNA and downregulation of inflammatory genes in activated endothelium* in vivo* [244]. Similarly, lipopolyplexes comprising imidazole/imidazolium lipophosphoramidate, histidinylated polyethyleneimine, and siRNA are used as efficient formulation for siRNA transfection [245]. However, for systemic delivery of siRNA and enhanced endosomal/lysosomal escape distearoyl phosphoethanolamine-polycarboxybetaine lipids are used [243]. Further, addition of polypropylene glycol to multiblock copolymer optimizes siRNA delivery [246]. However, tumor priming enhances siRNA delivery and transfection in intraperitoneal tumors [247] while O(6)-methylguanine-DNA methyltransferase-siRNA/liposome complex is administered by convection-enhanced delivery to rat and porcine brains [248]. Moreover different lipidic systems are used for* in vivo* siRNA delivery [249].

### 6.8. Colloidal Drug Carriers

Colloidal drug carriers such as liposomes and nanoparticles are used to improve the therapeutic index of both established and new drugs by modifying their distribution applications (Table 3) [250]. These are proved to be better drug delivery systems [178] because indirectly they increase drug efficacy, by reducing their toxicity [250]. Colloidal drug carrier systems such as micellar solutions (microemulsions), vesicles, and liquid crystal dispersions, as well as nanoparticle dispersions consisting of small particles of 10–400 nm diameters in size, are used to optimize drug loading and release. These show long shelf-life and low toxicity [178]. Similarly, microemulsions are used to deliver new classes of active molecules, such as peptides and proteins, genes, and oligonucleotides. The incorporated drug participates in the microstructure of the system, but its structure is affected due to molecular interactions, especially if the drug possesses amphiphilic and/or mesogenic properties [178]. These systems form spontaneously combining appropriate amounts of a lipophilic and a hydrophilic ingredient, as well as a surfactant and a cosurfactant. They may also offer alternative modes for more conventional drugs, such as highly hydrophobic small molecules. The formation of a ME is accompanied by a significant increase in the interfacial area. The required very low interfacial tension cannot be realized by only one surfactant. The additionally used cosurfactant penetrates the amphiphilic interfacial layer and increases its curvature and fluidity [251, 252]. Two types of MEs are differentiated: bicontinuous ones and MEs with droplet like structure. The droplet structures are forming depending on the major compounds water-in-oil (w/o) and oil-in-water (o/w) MEs having colloidal phases in the range of 10–100 nm which are colloidal structures such as solubilized micellar systems. These are also known as swollen micelles. In addition, colloidal or particulate carrier systems widely interact with cell microenvironment and are widely used as cargo carriers in vaccine therapies of CNS pathogens (Table 3). More specifically, polymeric particulate systems can be used as effective delivery tool by providing control over spatial and temporal distribution of cargos after systemic or localized administration along with enhancing their stability profile [253]. Curcumin-loaded solid lipid nanoparticles can control drug release and improve bioavailability. These showed high drug entrapment efficiency and loading capacity [254]. Further there is a need for optimizing different drug delivery systems for better therapeutic aids to the patients [255].

### 6.9. Liposomes

Liposomes are widely used as carriers or delivery vehicles for therapeutic agents/drugs to send them at specific sites inside human body. These are vesicles of phospholipids that form spontaneously in solutions and are capable of trapping dissolved particles in solutions. As most of the drugs do not cross the BBB, hence for its delivery, liposome technology is proved highly applicable (Figure 6). Further, advancements in liposomal drug delivery have produced long circulating and highly stable drug formulations. However, by making numerous improvements a number of liposome-based formulations are being made which effectively work as drug carriers. Liposomes are biodegradable liberating the charged molecules slowly when they degrade in the organism. Many of them are commercially available and some are in the developing phase and are undergoing clinical trials. These formulations can minimize systemic exposure, after transportation of drug and its biodistribution in target organs, cells, or compartments within the cells with or without expression of target recognition molecules on liposome membranes [245]. However, to increase the clinical use of liposome, drug interaction and liposome deposition mechanism lipid-drug association is more feasible for making the drug more accessible in to the brain for various therapies. Moreover, liposomal drug delivery methods are widely used for brain tumor and antimicrobial therapeutics. These are also highly applicable for gene transfer into cells that could be obtained by appropriate selection of the gene transfer vector and mode of delivery.

Liposomes are lyotropic liquid crystals composed of relatively biocompatible and biodegradable materials and consist of an aqueous core entrapped by one or more bilayers of natural and/or synthetic lipids. These are composed of natural lipids and are biodegradable, biologically inert, and weakly immunogenic and produce no antigenic or pyrogenic reactions and show limited intrinsic toxicity. Liposomes are versatile drug carriers, which can be used to control retention of entrapped drugs in the presence of biological fluids (Table 3). These showed controlled vesicle residence in the systemic circulation in the body and enhanced vesicle uptake by target cells. Therefore, drugs encapsulated in liposomes are expected to be transported without rapid degradation and minimum side effects to the recipients. Due to more dispersive property and stability in both acidic and basic conditions, liposomes are considered well-established carriers and have wider applications in biomedicine and food industry [256]. Unfortunately, therapeutic efficacy of liposomes remains limited due to the slow diffusion of liposomal particles within the tumor and its limited release or uptake of drug in many cases [257]. However, reformulation of drugs in liposomes will provide an opportunity to enhance the therapeutic indices of various chemical agents mainly through the alteration of biodistribution (Table 3).

Liposomes and polymersomes are generally used as carriers for encapsulating compounds, in particular drugs for delivery. However, synthesis of nanoparticles with an emphasis on the use of self-assembled systems such as micelles, microemulsions, nanoemulsions, and liposomes can increase the drug distribution, bioavailability, and its targeted action [258]. Thus, for better chemotherapeutics liposomal drug carriers are used for controlled release of active drug formulations at a predetermined rate. However, for achieving more stable circulation liposomes are conjugated with carboxyl-terminated CRPPR peptide and nontargeted liposomes to enhance the drug delivery into tumors. It shows affinity for the receptor neuropilin-1 (NRP) is and expressed on both endothelial and cancer cells [257]. Similarly, carboxyl-terminated RXXR peptide, conjugated to liposomes retains long circulation, enhances drug binding and internalization and finally cut down toxicity [257]. However, for targeting of drugs many drug carriers like serum proteins, immunoglobulins, synthetic polymers, liposomes, niosomes, microspheres (Figure 6), erythrocytes, reverse micelles, pharmacosomes, and monoclonal antibodies are synthesized and used.

However, for delivery of anticancer drugs to the target site and for more effective treatment specific delivery systems are generated. Further, anticancer drugs are designed that work with mild hyperthermia-mediated triggering and tumor-specific delivery. Hence, thermosensitive liposomes [258] are made by using thermosensitive polymers [259]. Further, targeted and ultrasound triggered drug delivery systems are made in which liposomes are comodified with cancer cell-targeting aptamers and thermosensitive polymers [259]. Further, to enhance the thermosensitive drug release, elastin-like polypeptide (ELP) is incorporated (thermally responsive phase transition peptide) into the dipalmitoylphosphatidylcholine- (DPPC-) based liposome surface [260]. Additionally, cyclic arginine-glycine-aspartic acid (cRGD) binds to *α*_*v*_ *β*_3_ integrin, which is overexpressed in angiogenic vasculature and tumor cells, and was introduced on the liposome. Moreover, ELP-modified liposomes with the cRGD targeting moiety were prepared using a lipid film hydration method, and doxorubicin (DOX) was loaded into the liposome by the ammonium sulfate-gradient method. The cRGD-targeted and ELP-modified DOX-encapsulated liposomes (RELs) formed spherical vesicles with a mean diameter of 181 nm. The RELs showed 75% and 83% DOX release at 42°C and 45°C, respectively. The stability of RELs was maintained up to 12 h without the loss of their thermosensitive function for drug release. These stable, target-specific, and thermosensitive liposomes are promisingly used to enhance therapeutic efficacy (Figure 6) of anticancer drugs and are applied along with a relevant external heat-generating medical system (Table 3, Figure 4) [260].

Similarly, for the treatment of blood malignancies targeted particulate drug delivery systems are developed. These could employ targeted liposomal formulations for B cell malignancies [261]. For example liposomal encapsulation of antineoplastic agents such as AD 198 has been made that is proved to be superior to doxorubicin [261]. Similarly, PEG-coated irinotecan cationic liposomes have shown better therapeutic efficacy against breast cancer in animals [262]. Furthermore, many improved liposomal formulations such as loaded ethosomes to carry drugs across human skin [263], terbinafine HCL liposomes for cutaneous delivery [264], and curcumin-loaded cationic liposomes are prepared for various cancer therapies [265]. Similarly, novel transferrin embedded fluorescent magneto-liposome nanoformulations were made which have shown enhanced blood brain barrier transmigration [266]. Further, liposomes comodified with cholesterol anchored cleavable PEG and octaarginines were made for targeted drug delivery [267]. In addition, PEGylation improves the receptor mediated transfection efficiency of peptide-targeted, self-assembling, anionic nanocomplexes [268]. Further, electrostatically driven complexation of liposomes with a star shaped polyelectrolyte was used to have low toxicity multiliposomal assemblies [269]. However, to enhance surface functionalization different anchoring lipids were used via Staudinger ligation [270].

However, for effective cancer therapeutics nanoscale drug delivery systems such as liposomes, polymers, and other nanoparticles were developed that provide potential solutions and are currently in use. Moreover, all current liposomal drugs were evolved from a number of drug designs and strategies tested in the laboratory for improved biodistribution within the body. Moreover, liposomes afford a unique opportunity to deliver the drugs into cells by fusion or endocytosis mechanism and practically any drug that can be entrapped into liposomes irrespective of its solubility. However, *α*-helical peptides synthesized* de novo* induce aggregation of various kinds of cells by focusing on physicochemical properties such as hydrophobicity, net charges, and amphipathicity. Further, liposomal formulations having cell-aggregating peptides lead to aggregation of living cells without cytotoxicity [271]. Moreover, peptide hydrophobicity is the key factor that determines capabilities for cell aggregation while peptide net charges contribute to nonspecific electrostatic interactions with cells. These amphipathic peptides tend to exhibit cytotoxicity such as antimicrobial activity and hemolysis, which are competitive with cell-aggregation capabilities. In addition, aggregation of artificial anionic liposomes appears to be mainly determined by electrostatic interactions.

However, drugs with wide variations in lipophilicities can be encapsulated in liposomes either in the phospholipid bilayer, in the entrapped aqueous core, or at the bilayer interface. Because in liposomes water soluble and fat-soluble medications are trapped inside two different layers and one end remains inside the water while another end or the drug remains trapped inside aggregation of hydrophobic ends. However, in most of the liposomes one end of each molecule is water soluble, while the opposite end is water insoluble [272]. But in few cases liposomes are found to attach to cellular membranes and fuse with them and simultaneously release drugs into the cell [273]. Interestingly, these are internalized by phagocytic cells, and phospholipid walls are acted upon by lysosomes, and the medication is released. However, sometimes the large size of the liposomes produces microembolisms that gave a false impression of brain uptake [251]. Therefore, for solving the brain drug delivery problem, lipidization of the drug should be made. For this purpose, a water soluble drug should be converted into a lipid soluble drug by changing the functional groups. However, to diffuse the restriction imposed by BBB conversion of water soluble drug into lipid soluble prodrug could be a complete solution [274].

Liposomes are better suited for assessing their targetable properties because of the ease of modifying their surface when compared to other drug carriers such as nanoparticles [206, 275] and microemulsions [276, 277] (Table 3, Figure 6). However, various approaches have been attempted to increase drug accumulation, internalization, and therapeutic efficacy [257]. Therefore, various biodegradable materials are used to form liposomes for different purposes. A palmitic cationic liposomal* in situ *gel protects from external compounds and keeps the drug intact in its natural form [278]. Similarly, polyglycerol coating to plasmid DNA lipplex is used for the evasion of the accelerated blood clearance phenomenon in nucleic acid delivery [279]. Further, to achieve targetable carrier properties various noncovalent associations of cell-specific antibodies with liposomes are being made [280]. Similar, covalent attachment of poly- and monoclonal antibodies to the liposomes [281, 282] and coating of liposomes with heat aggregated immunoglobulins M (IgM) are also done [283]. Similarly, natural [284] and synthetic [285] glycolipid [286, 287], glycoprotein bearing liposomes [288], and transferrin coated paclitaxel loaded [289], lysozyme liposomes [281], and neuroipilin-1-targeted liposomes were made to enhance delivery and bioefficacy of drugs [257]. More specifically, compounds entrapped into the liposomes are protected from the action of external media, particularly enzymes [290] and inhibitors [291]. However, RGD-lipid conjugate-modified liposomes [292] are used for enhancing siRNA delivery in human retinal pigment epithelial cells [293] (Table 3).

However, liposomal nanoparticles are proved to be multifunctional tools [258] to carry various drugs for cancer therapy. These liposomal siRNA nanocarriers are also used in tumor therapy [239, 246] while enhanced endosomal/lysosomal escape by diesteryl phosphoethanolamine-polycarboxybetaine lipid is used for systemic delivery of siRNA [294]. Similarly, cationic liposome mediated delivery of FUS1 and hil12 is used to treat human lung cancer. These are also used as transfecting agents of DNA in gene therapy [290]. Moreover, endothelial targeting of liposomes encapsulating SOD/catalase EUK-134 alleviates acute pulmonary inflammation [295]. However, to optimize the application, polymeric core-shell [296], amphiphilic block copolymer [273], molecular imprinted polymers are used for preparing advanced drug delivery devices [297]. Similarly, supramolecular drug delivery systems are used for membrane permeability with bacterial porins [298] and bioadhesive microspheres are used for controlled drug delivery system [299]. Moreover, all existing liposomal delivery systems are experimentally confirmed which can transfer sizable amount of drug. This will optimize drug action and target specificity in diseased tissues in particular region of brain. Moreover, efforts have been made to increase the specificity of carriers to carry drugs to the target organs mainly to cells or within various cellular compartments. However, lipidization of the drug functions is considered as a noninvasive approach to solving the toxicity related brain drug delivery problem. However, to optimize the drug action water soluble drug compound could be made lipid soluble by making slight change in its functional groups. This could uplift transport restriction by conversion of water soluble drug into lipid soluble prodrug [300]. However, polysorbate 80, a detergent, is used to disrupt the BBB, which also act as a drug stabilizing agent and attributes detergent effects to nanoparticles that assist in drug delivery (Figure 4). But a large size of the liposomes produces microembolisms and obstructs the drug uptake and gives a false impression [251]. Further, modular organization of immunoliposome technology enables a combinatorial approach in which a repertoire of monoclonal antibody segments can be used in conjunction with a series of liposomal drugs to yield a new generation of molecularly targeted agents (Table 3) [301].

### 6.10. Micelles

Micelle is an aggregate of surfactant molecules dispersed in a liquid colloid. A typical micelle in aqueous solution forms an aggregate with the hydrophilic “head” regions in contact with surrounding solvent, sequestering the hydrophobic single-tail regions in the micelle centre. This phase is created by the packing behavior of single-tailed lipids in a bilayer. It is formed by filling in volume of the interior of a bilayer and area per head group forced on the molecule by the hydration of the lipid head group. Micelles are formed if one of the two fatty acyl chains is removed from the phosphoglycerides by hydrolysis forming a lysophospholipids. It forms a normal-phase micelle or oil-in-water micelle (Figures 4 and 6). Micelles are rarely formed from natural phosphoglycerides whose fatty acid side chains are too bulky to fit into the interior of a micelle. Normally, in aqueous solutions, common detergents and soaps form micelles that behave as tiny ball bearings thus giving soap solutions thin slipper fed and lubricating prospective. Naturally, biomembrane contains cholesterol, glycolipids, and proteins but these possess hydrophobic core that separates two aqueous solutions and acts as a permeability barrier. More specifically in phospholipids and sphingolipids and hydrocarbons tails of fatty acids side chains are hydrophobic while heads are strongly hydrophilic. Moreover, phospholipids are amphipathic in nature, are quite interactive, and form a sealed compound surrounding an internal aqueous space. Hence, a suspension of phospholipids upon its mechanical dispersion in aqueous solution aggregate to form spherical micelles, liposomes and phospholipid bilayer. The phospholipid bilayer is the basic structural unit of nearly all biological membranes (Figure 6).

Contrary to this inverse micelles have the head groups at the centre with the tails extending out and forming water-in-oil micelle. Micelles are approximately spherical in shape but its other shapes such as ellipsoids, cylinders, and bilayers are also possible. Both shape and size of a micelle are a function of the molecular geometry of its surfactant molecules and solution conditions such as surfactant concentration, temperature, pH, and ionic strength. However, micelle chemical composition, total molecular weight, and block length ratios can be easily changed, which allows control of the size and morphology of the micelles. Further, functionalization of block copolymers with cross-linkable groups can increase the stability of the corresponding micelles and improve their temporal control [302, 303]. However, micelles formed by self-assembly of amphiphilic block copolymers (5–50 nm) in aqueous solutions have wider drug delivery applications [290]. These micellar structures physically entrapped the drug and transported it to the target area and released required concentrations. It exceeds due to intrinsic water solubility. Further, the hydrophilic blocks form hydrogen bonds with the aqueous surroundings and form a tight shell around the micellar core. As a result, the contents of the hydrophobic core are effectively protected against hydrolysis and enzymatic degradation [302].

Polymeric micelles are new drug carrier systems, which are used for drug targeting of anticancer drugs to solid tumors. It is a macromolecular assembly composed of an inner core and an outer shell and most typically is formed from block copolymer that is suitable for encapsulation of poor water soluble, hydrophobic anticancer drugs. Polymeric micelles are of nanorange in size and show stability and longevity* in vivo* that is why these areused for targeted delivery at the tumor sites by passive mechanism where they show enhanced permeability and retention effect. Other characteristics of polymeric micelles such as separated functionality at the outer shell are useful for targeting the anticancer drug to tumor by active mechanisms [303]. Polymeric micelles are considerably more stable than surfactant micelles and can solubilize substantial amounts of hydrophobic compounds in their inner core. Polymeric micelles also enhance pharmacological activity of drugs [304] and show potential medical applications, especially in cancer chemotherapy [305] (Figure 6, Table 3). Due to their hydrophilic shell and small size polymeric micelles accumulate in tumoral tissues and persist for longer duration [301, 306]. Polymeric micelles can be conjugated with many ligands such as antibodies fragments, epidermal growth factors, *α*_2_-glycoprotein, transferrin, and folate to target micelles to cancer cells. However, polymeric micelle could deliver drugs by both passive and active mechanisms [303, 304]. These successfully obstruct tumor angiogenesis and find potential targets of anticancer drugs [303].

## 7. Cellular Mechanisms for Drug Targeting

BBB restrict entry of most of the biomolecules mainly proteins, peptides, carbohydrates, and vaccines. Hence, delivery of therapeutic peptides and proteins to the central nervous system is the biggest challenge for development of more effective neuropharmaceuticals [307]. BBB is impermeable to most molecules and most of the proteins found in the plasma are not able to cross the blood brain barrier because of their size and hydrophilicity. But few peptide hormones which regulate body metabolism and normal functions of catabolites as both insulin and transferrin, concentration varies in plasma and uptake of these peptides in the brain is greater than expected based on their size and lipid solubility. These are carried to the brain by specific transport processes mainly membrane bound efflux pumps and channels. The major transport mechanism which carries proteins and hormones is receptor mediated transcytosis. However, therapeutic agents may reach to the target sites at intracellular locations. The brain capillary endothelial cell is highly enriched in receptors for these proteins, and following binding of protein to the receptor, a portion of the membrane containing the protein-receptor complex is endocytosed into the endothelial cell to form a vesicle. Although the subsequent route of passage of the protein through the endothelial cell is not known, eventual release of intact protein on the other side of the endothelial cell is highly useful because blood brain barrier is impermeable to these molecules [307]. During delivery process a portion of compound was lost due to ineffective partitioning across the membrane. Hence, partitioning across the membrane is widely concerned with polarity, lipophilicity of molecules that attributes easy passage across the membrane. However, amphiphilic derivatives of a peptide are easily delivered into the brain. These are designed to self-assemble into nanofibre which in the active peptide epitope is tightly wrapped around the nanofibre core [307]. Recently, several neuroprotective proteins and peptides of potential therapeutic value have been designed that showed effective and safe transcapillary movement into the brain. Therefore, most promising drug delivery through brain capillaries is only possible by augmentation of pinocytotic vesicles because it is a fully noninvasive method. This is a cellular mechanism which assist in delivery of large molecules of neurotherapeutic potential by conjugating them with peptidomimetic ligands. Later on these molecules bind to selected peptide receptors, which internalize and transport it in small vesicles across the cytoplasmic brain capillary barrier. These conjugates are found functionally active and effective in animal models of neurological disease. Similarly, neurotrophin, a brain derived neurotrophin, a brain-derived neurotrophic factor easily passes through BBB and has great therapeutic value. Interestingly short peptides with hydrophilic nature have shown favorable safety profiles in brain and found neuroprotective after come across the BBB. However, exogenous recombinant human erythropoietin was proved to be beneficial in treating global and focal cerebral ischemia and reducing nervous system inflammation in experimental animals. Moreover, other than neuroprotective compounds monoclonal antibodies are also used to pass through BBB by receptor mediated endocytosis mechanism. Similarly, metallothionins a superfamily of highly conserved, low molecular weight polypeptides play a significant role in the regulation of concentration of essential metals which are also internalized by receptor mediated endocytosis. However, variable efficiencies of endocytosis mechanisms, such as intracellular trafficking and release of therapeutic agents in to the cytoplasm, are important aspects in drug delivery and therapeutic potency. There are many possibilities after diffusion and translocation of the therapeutic agents. These remain susceptible targets of certain catalytic enzymes or physically partition into the nucleus or in any other suborganelles that may also alter its actual activity. Further, excess delivery of therapeutic agents may create a competitive problem to some other biomolecules that may hinder normal functions of cells, cellular organelles, enzymes, and signaling molecules. In addition, metabolic wastes may also over burden the cell cytoplasm that inhibits so many normal cellular functions and give rise to drug induced adverse effects. However, use of nanoparticles may solve this problem due to controlled release of drugs in required quantity. These can easily cut down concentration of metabolic waste materials by masking the therapeutic agents from its biological environment. Nanoparticles allow controlled (sustained) drug release from the matrix, determine required bioavailability, and show reduction of the dosing frequency. These are proved to be most successful drug carriers due to their high stability, high carrier capacity, and feasibility of incorporation of both hydrophilic and hydrophobic substances into brain or inside cells. These also show feasibility to deliver drugs by following variable routes of administration, including oral application, and inhalation.

Normally, two mechanisms are employed to ascertain the internalization of biomolecules; mainly liquids are poured in by pinocytosis and solids by phagocytosis. However, there is carrier mediated delivery of drugs by nanoparticles and these are ingested by cells from the medium or from any microenvironment surrounding the cell. However, nanoparticles are pouring in by receptor mediated endocytosis that could operate by membrane manipulation to envelope and allow materials to absorb inside. Therefore, it is clearer that nanoparticles get inside the cells by three different mechanisms, that is, phagocytosis, pinocytosis, and receptor mediated endocytosis. Furthermore, phagocytosis is associated with few cell types such as macrophages, neutrophils, and dendritic cells which can absorb materials of micrometer in size, that is, 10 *μ*m in diameter. Similarly, pinocytosis is a universal mechanism which occurs in all cell types and it delivers different types of liquids having a submicron size and substances in solution inside the cell. More specifically, larger sized nanoparticles are taken up by the cell by phagocytosis, while smaller ones are absorbed by pinocytosis and most of them are ingested by all cell types. Therefore, both types of nanoparticles have important but separate advantages. Furthermore there is another mechanism which is known as absorptive-mediated transcytosis that is especially used to traverse polycationic proteins and lectins. This is a nonspecific process in which, proteins are adsorbed on the endothelial cell membrane based on charge or affinity for sugar moieties of membrane glycoproteins. Its subsequent transcytotic events are probably similar to receptor mediated transcytosis. However, overall capacity of absorptive-mediated transcytosis is far greater than that of the receptor mediated endocytosis because the number of receptors present in the membrane does not limit it. Thus, cationization may provide a mechanism for enhancing brain uptake of almost any protein.

## 8. Conclusion

Because of limits imposed by structural barrier (BBB) the delivery of therapeutic drugs to brain remains a challenging task to treat patients suffering from tumors, virus generated neuronal infections, and neurodegenerative diseases. However, for proper medication of patients several approaches have been developed and used for direct and indirect delivery of drugs to the brain. But, sometimes direct injections or convection-enhanced delivery of drug or cerebrospinal fluid or intranasal delivery creates problems to the patient or remains unsuccessful. These approaches are proved to be very much unsafe, highly invasive, and short lasting. Therefore, targeted molecular based therapies are developed for treatment of brain tumors that could deliver the antitumor drugs to the target sites and stop aberrant signaling pathways in the brain. Further, vascular route should be improved to make it more promising for drug delivery to the brain because it allows a widespread diffusion of the infused drug throughout brain and covers a large surface area. Hence, drugs that could find their way through nonbarrier regions will be preferred to lower down the risk of neuronal injuries, nondelivery, and therapeutic failures. Hence, there is a need to generate new nanosized carrier vehicles that could easily pass through systemic microvascular beds found in blood capillaries and endothelial cells for safe delivery of pharmaceuticals. Therefore, natural formulations should be developed that could passively pass through discontinuous tight junctions or with the help of plasmalemmal vesicles and windows occurring in endothelial cells. Therefore, novel strategies that could overcome the intrinsic limitations of the BBB are highly desirable.

Further, to lower down the risk of nanoparticle generated cytotoxicity and invasiveness, biodegradable biomaterials should be used to minimize toxic effects in the brain. Biomaterials used for making nanoparticle should be biocompatible and must have very short half-life. Therefore, biodegradable polymers like polylactic acid, polycaprolactone, poly(lactic-co-glycolic acid), the poly(fumaric-co-sebacic) anhydride chitosan, and modified chitosan, as well as solid lipids, should be used to prepare nanoparticles. Further, to reduce the drug toxicity and to minimize its adverse effects simpler drug conjugates like doxorubicin can be attached. Moreover, active drug molecules can be coupled to a desired protein or peptide that increases its circulating life, solubility stability and antigenicity. Further, various nanoprodrugs should be prepared by using spontaneous nonemulsifiable biodegradable antioxidants and vitamins to enhance therapeutic efficacy of drugs in the oxidative tumor microenvironment [308]. Moreover, modification of nanoparticle surface with covalently attached targeting ligands or by coating with certain surfactants is essential for receptor mediated uptake or adsorption of specific plasma proteins after injection in human. A proper avidity is also required for nanoparticles to reach the brain parenchyma, and it should be consistent with transcytosing antibodies that bind to TfR [309]. It can be used for delivery of a great variety of drugs including anticancer, analgesics, cardiovascular, protease inhibitors, and several macromolecules into the brain after intravenous injection in animals. In addition, chimeric peptides, lipids, and beta-cyclodextrin carriers can be used as colloidal drug carriers [169]. Further, for systemic administration transferrin (Tf) bound gold nanoparticles are proved highly useful for transport of therapeutic agents into the brain. Therefore, advanced drug delivery systems are to be developed for transport of potential biopharmaceuticals to treat CNS related disorders, pathogenesis, and neoplasticity. More specifically, among all existing drug delivery methods nanotechnology holds great promise for a noninvasive therapy of brain tumors and other CNS diseases.

Moreover, noninvasive methods like contrast enhanced microbubble ultrasound should be preferred for drug delivery because generation of microbubbles leads to drug perfusion enhancement in nervous tissue. It also directly increases amount of drug delivered into the brain. Further, receptor mediated delivery systems are used for delivery of proteins/peptidal drugs. However, methods such as receptor mediated endocytosis, loaded microbubble enhanced focused ultrasound, and cholesterol mediated cationic and solid lipid nanoparticles delivery system, SiRNA delivery system, colloidal drug carriers, liposomes, and micelles should be reinvestigated for their further advancements to enhance the targeted drug delivery of therapeutic agents. Further, safer and noninvasive methods such as micelles formed from natural phosphoglycerides are used to deliver the drug. Similarly, various types of liposomes such as PEGylated liposomes, niosomes, and aquasomes are specially used for peptidal drug delivery. Further, combination therapies are to be developed for tumor ablation [144] and inhibition of cancer associated mutations by peptide masking [310]. Further, intracarotid infusion of bradykinin (BK), nitric oxide (NO) donors, or agonists of soluble guanylate cyclase (sGC), and calcium-dependent potassium K(Ca+) channels enhance drug delivery into the brain. These were found to be more effective and safer to treat tumor patients [21]. Further, for targeted drug delivery, a series of amino acids dipeptide diester prodrugs of NO donating oleonotic disruptive are to be designed. These should be practiced to find an appropriate solution of CNS related pathogenicity and neurodegenerating diseases. Similarly new hydrogels should be prepared for transdermal delivery of drugs to treat skin and dermal cancers [311]. Furthermore, fine nanocarriers/vehicles such as membrane transporters and ABC cassettes, molecular drug transporters, or delivery vehicles are to be developed. In addition natural transporters are favored to support transport of drugs, nourishments to maintain vital brain functions. In addition, role of various drug transporters and permeablitizers must be reinvestigated.

Therefore, for active distribution of drug, its carrier loading, targeting, and transport foolproof drug delivery systems are to be developed. In addition, its interactions with biological barriers should be properly investigated in experimental animals as well as in* in vitro* systems. Moreover, advanced methods are to be developed for easy delivery of healers, peptides, proteins, growth factors, vaccines, and antibodies for treatment of CNS diseases and disorders. Further, there seems to be an instant need of new smaller pharmaceuticals having target specific designs. Hence, a long term planning is required for stepwise upgradation of pharmaceuticals and to have design of highly absorbable drugs. Further, technologically upgraded simpler drug delivery systems are to be developed for making much faster strategic defense against different types of tumors, cancers, disorders, and viral diseases. It may not only help to deliver the pharmaceuticals but also to assist in finding new signaling pathways that may help in diagnosis, assimilation of drugs, and its active functions in infectious tissues. Hence, new absorbable drug designs having nanoscale particle size and showing high target specificity and transcellular signaling should develop. These new drug candidates must be pretested* in vitro* systems to find appropriateness of drug action and to authenticate the behavior of biopharmaceuticals. Therefore, to fulfill drug delivery tasks a better understanding is required among clinicians and immunologists for starting new research initiatives to make landmark innovations in the field of pharmacology, molecular biology, and clinical therapeutics of CNS related diseases. Hence, strong recommendations are being made to upgrade pharmaceutical technologies by making collaborative research efforts to develop/explore new innovative methods for safer drug delivery. It is only possible by making advances in nanobiotechnology and biomaterial sciences to extend the therapeutic use of pharmaceuticals to cure neurological diseases and CNS impairments. Further, biomedical researchers should increase the spectrum of pharmaceuticals by carrying them to targeted locations by improving the endothelial transport methods. There is an essential need of new more innovative noninvasive and nontoxic delivery methods to find quick and easy solution of neurodegenerative and neuropathological diseases of CNS.

## Figures and Tables

**Table 1:** Different types of drug delivery methods used for CNS protection and tumor therapy.

Delivery vehicle/s	Route of transfer	Method	TM	Advantages	Disadvantages	Applications	References
Colloidal nanoparticles	Intranasal	Direct	DF∗	Noninvasive and safe, neuroprotective	Poor release of drug	Use for delivery of local ailments of cold cough	[34]

Lipid nanoparticles	Intraventricular	Direct	DF	Enhance drug efficacy, neuroprotective	Toxic to membranes	Rhinitis and ischemic brain injury, for tumor, and global ischemia	[34]

Direct injection	Intraventricular	Direct	DF	Less toxic, impose fewer side effects, and neuroprotective	Invasive and toxic	Use for delivery of pain medication within the CSF	[49]

Prodrugs	Oral or intranasal	Direct/indirect	DF	Tissue targeted delivery of lipophilic molecules, safe	Poor biological activity	Largely used to treat neuronal diseases	[25]

Peptide masking	Injection	Direct	DF/RMT∗∗	Cholesteryl group traverse the drug through BBB	Poor biological activity	Multiple sclerosis, cancer and tumor	[28]

Proteins	Transcapillary	Indirect	RMT	Less toxic, effective and safe, and neuroprotective	Difficult transport	Effective against cerebral ischemia, neurorepair after trauma	[58]

Chimeric peptides	Transcapillary^$^	Indirect	RMT	Targeted drug delivery, stable during transcytosis	Less permeable	Effective in treatment of various neurodegenerative diseases	[59]

Radionuclides^#^	Transcapillary	Indirect	DF/contact	Tumor detection and ablation, low dose, and neuroprotective	Necrosis and lesions	Neuroimaging of brain and neuroendocrine tumors	[60]

LMEFU∗∗∗	Intraventricular	Indirect	DF	Noninvasive, distribute drug reversibly, and neuroprotective	Cause structural injury	Cancer and tumor therapeutics, neurodegenerative diseases	[61]

Proline rich peptides	Transcapillary	Indirect	DF/RMT	Less toxic, safe and effective, and neuroprotective	Catalytically unstable	Use for treatment of cerebral infections neurocognitive disorders	[62]

*DF: diffusion, ∗∗RMT: receptor mediated transcytosis, ∗∗∗loaded microbubble enhanced focused ultrasound, ^$^fluorescent protein,^#^radiopharmaceuticals, and TM: transport mechanism.

**Table 2:** Different types of inorganic nanoparticles, their uses, and application in biomedicine.

Inorganic nanoparticles	Composition	Applications	Advantages
Chitosan-nanoconjugated hormone nanoparticles	Chitosan and hormone	Deliver nontoxic, polynucleotide pharmaceuticals to neurocompartments	Show low immunogenicity

Insulin nanoparticles	Polymeric nanoparticle-cross-linked/beads	Oral delivery of insulin, imitates the production of insulin by pancreatic islet cells	Overcome cancer drug resistance, targeted treatment across barrier

Smrho protein loaded chitosan	Coated with sodium alginate or alginate	Oral vaccination, stable and fine target accessibility, and good immunization against *S. mansoni *	Great stability and ease of target accessibility, immunostimulatory

Chitosan-sodium lauryl sulfate nanoparticles	Anionic surfactant sodium lauryl sulfate	Oral delivery of insulin, biodegradable, stable in simulated gastric fluids, and bioavailability	Improve insulin oral bioavailability

Chitosan-Pluronic nanoparticles	Chitosan and Pluronic F-127	Efficient oral formulation for colon cancer treatment	Effective delivery system with few side effects

Chitosan-DNA nanoparticles	A complex coacervation of DNA, chitosan, and sodium sulfate	Protect the encapsulated plasmid and increase transfection efficiency	Better loading, release, and cell uptake

Lecithin/chitosan nanoparticles	Chitosan and lecithin colloidal suspension	Progesterone delivery, model lipophilic drug, and shows good encapsulation efficiencies	Transdermal delivery of melatonin, biocompatible

Chitosan-coated iron oxide nanoparticles	Fe_3_O_4_ nanoparticles as cores and chitosan (CS)	Noncytotoxic, PEG-CS-Fe_3_O_4_ as a stable magnetic targeting drug carrier in cancer therapy	Anticancer effect against human ovarian cancer cells, target integrin rich tumor cells

FVIII-chitosan nanoparticles	DNA polyplexes composed of chitosan and factor VIII DNA	Oral delivery of a nonviral gene carrier, hemophilia A gene therapy	Nonviral delivery for gene medicine applications, delivery system practical for hemophilia A gene therapy

PEGylated chitosan-modified	Lipid-based poly(ethylene glycol) (PEG)	Nontoxic biodegradable, oral, and dermal applications, improve the efficiency of the drug	PEGylated chitosan prolonged the retention time of the nanoparticles in the circulatory system and improved the bioavailability of cyclosporin A

mPEG-PLA Cyclosporin A-loaded	Polymeric micelles based on monomethoxy poly(ethylene glycol)-b-poly(d,l-lactic acid), (mPEG-PLA)	Spatial distribution of the drug within the nanoparticles	Improve the oral bioavailability of poor immune response

mPEG-PLA Cyclosporin A-loaded	Water soluble cyclosporin A (CyA) affected the intestinal P-gp efflux pumps	Good candidate for oral delivery of poorly soluble drugs	Stable and monodisperse nanoparticles (NPs) in aqueous suspension

Chitosan PGA nanoparticles (PLGA NP)	Polylactic-co-glycolic acid incorporated nanoparticles	Capacity in repairing and regenerating wounded and dysfunctional tissues	Targeted, highly effective and safe treatment of lung cancer.

Thiolated chitosan nanoparticles	A core of polymethyl methacrylate surrounded by a thiolated chitosan	Longer half-life, oral drug delivery system for anticancer drugs	Potential enhancer buccal delivery of insulin, tensile strength, and bioadhesion force

Beta cyclodextrin carries	Ammonium beta cyclodextrin, (Ch-GSH-pMMA)	Anticancer drug delivery vehicles	Biocompatible, less toxic

Quaternary ammonium *β*-cyclodextrin (QA*β*CD)	Ammonium *β*-cyclodextrin	Carrier for doxorubicin (DOX), and hydrophobic anticancer drug, across the BBB	Great potential in safely and effectively delivering DOX and other therapeutic agents across the BBB.

*β*-Cyclodextrin inclusion complexes	*β*-Cyclodextrin (*β*-CD), encapsulation	Delivery of neuroprotective drug	Form inclusion complexes which are a promising formulation for melanoma treatment, transdermal delivery of drugs

Amoxicillin *β*-cyclodextrin	Amoxicillin and *β*-lactam cyclodextrins of different sizes	Low toxicity and low pharmacological activity, protect drug molecules from biodegradation, increased drug transport	Orally administered sustained release formulation for the treatment of peptic ulcers

PLGA nanoparticles poly(lactide-co-glycolide)	Poly(lactide-co-glycolide)(PLGA), a biodegradable polyester	Anticancer enhanced drug delivery to tumor cells, higher efficacy, and fewer side effects	Antibody conjugated ICG-DOX-PLGA nanoparticles have potential for combinatorial chemotherapy and hyperthermia

Lansoprazole-loaded nanoparticles	Lansoprazole-loaded Eudragit RS100 nanoparticles (ERSNP-LPZ) as well as poly(lactic-co-glycolic acid)	Sustained and prolonged drug delivery	Novel lansoprazole-loaded nanoparticles for the treatment of gastric ccid secretion-related ulcers

Nanocrystals	Aggregates of molecules, crystalline form of drug	Better biological distribution and bioavailability	Reduce toxic effect of drug

Magnetic nanoparticles	Super paramagnetic iron oxide particles display large magnetic moments in a magnetic field	Targeting tumor cells	Induction of maturation on dendritic cells, via NF-*κ*B signaling pathway

Iron oxide nanoparticles	Ferromagnetic iron oxide nanoparticles and maghemite (y-Fe_2_O_3_) and magnetite (Fe_3_O_4_) nanoparticles	Sonochemical decomposition of iron pentacarbonyl, target integrin rich tumor cells	*In situ* forming hybrid iron oxide-hyaluronic acid hydrogel for magnetic resonance imaging and drug delivery

*Metallic *			

Silver nanoparticles	Ag^+^-NOM-Iron(II, III) systems	Antibacterial activity controlled release of drugs, proteins, and DNA	Silver nanoparticles crossing through and distribution in the blood brain barrier *in vitro*, glioma treatment

Gold nanoparticles	Gold solid nanoparticles	Good biocompatibility and easy surface modification utilize the GNPs as multifunctional probes tumor—specific targeting moieties, controlled release of drugs, proteins, and DNA, and used in photoacoustic tomography	Encapsulation, biosensing and imaging, when decorated with oligo(ethylene glycol) thiols show increase in surface charges and interactions with proteins in solution

*Nanoshells *			

Silica nanoparticles	Coexistence of hydrophilic surface silanol (–Si–OH) and deprotonated silanol (–Si–O–) groups photostable	Nontoxicity and good biocompatibility prepared by sol-gel method, 3-aminopropyltrimethoxysilane, allyltrimethoxysilane	Easily cross the blood brain barrier, show higher drug delivery, and show transferring conjugation

*Ceramic nanoparticles *			

Layered double hydroxide nanoparticles	Coprecipitation of mixed salts, 40–300 nm.	Low cytotoxicity, biocompatibility	Delivery of anticancer drug incorporated in double layer, enhanced anticancer therapeutic efficacy

Calcium phosphate nanoparticles	Hydroxyapatite	Excellent biocompatibility, limited aggregation	Biocompatible, less toxic

Polysorbate-coated nanoparticles	Polysorbate	Transported across the capillary wall, improve the action of drug or any other pharmaceutical across the barrier	Mimic low-density lipoprotein (LDL), enhance drug delivery

ATP binding cassettes	Proteins	Protect against neurotoxicants and limit drug delivery, reduce xenobiotic efflux, rapid transportation of drug across the cell membrane, neuroprotective agent	Cerebral clearance of endogenous neurotoxic compounds

**Table 3:** Different types of organic nanoparticles, their uses, and application in biomedicine.

Organic nanoparticles	Composition	Applications	Advantages
Peptide-based nanoparticles	Ferritin protein cage nanoparticles family of proteins, 10–500 nm	Chemically or genetically modified, multifunctional probes for tumor imaging; ferritin is pH dependent, nanoparticles (NPs) decorated with transferrin (Tf)	Used for nasopharyngeal cancer-specific therapy

Lipid-based nanoparticle	Cholesterol mediated cationic solid nanoparticles 10–400 nm	Used for delivery of proteins and peptides and used for immune-stimulatory RNA adjuvant, cancer therapy, anti-viral agents, brain tumors.	High drug entrapment efficiency and loading capacity

Solid lipid nanoparticles	Colloidal 10–700 nm	Solid lipid nanoparticles can be used as colloidal drug carriers for various therapeutics, pharmaceutical alternative of liposomes and emulsions	Used to deliver drug orally, topically, or by inhalation

SiRNA delivery systems	SiRNA 5–40 nm	Used in malignant melanomas and cancer therapy	Suppress effects of oncogenes effective vehicles for delivery of PrP

Colloidal drug carriers	10–400 nm diameters in size microemulsions	Cargo carriers in vaccine therapies of CNS pathogens.	High drug entrapment efficiency and loading capacity

Liposome drug carriers	Closed spherical assemblies of amphiphilic delivery vehicles 10–700 nm	For therapeutic agents/drugs minimize systemic exposure, gene transfer vector, and mode of delivery, biocompatible and biodegradable materials, applications in biomedicine and food industry; liposomes can increase the drug distribution, bioavailability and its targeted action, anticancer drugs	Nontoxic biodegradable, prolong circulation of drugs

Magneto-liposomes phospholipid bilayers	50–100 nontoxic biodegradable, nonantigenic, low systemic toxicity, prolong circulation of drugs, controlled	Drug release cause pseudoallergic inflammation, controlled delivery of drugs in aqueous space within liposome, intercalated in to lipid bilayers, gene delivery	Nontoxic biodegradable, low systematic toxicity, controlled drug release

Micelles	An aggregate of surfactant molecules dispersed in a liquid colloid, micellar structures mainly core of block copolymer	Micelles physically entrapped the drug and transport it to the target area and release required concentrations, formed by two fatty acyl chains	Deliver large amount of drugs to cancer cells

Polymeric micelles	An aggregate of surfactant molecules dispersed in a liquid colloid, 10–800 nm	New drug carrier systems stability in plasma, longevity, cancer chemotherapy obstruct tumor angiogenesis	Potential targets of anticancer drugs

Carbon nanotubes	Cylindrical graphite sheets 1.5–5000 length and 0.5–20 diameter, traverse cell membrane as nanoneedles, thermal	Conductivity, target tumors. Insoluble in aqueous media, cytotoxic, poor incorporation capacity, targeted delivery of drugs, genes, vaccines, antibodies, and thermotherapy of tumors	Traverse cell membrane, show thermal conductivity, and target tumors

Quantam dots	Colloidal graphic sheets rolled in to single or multiwalled tubes <10 nm, predict emission frequencies, brighter and stable signal intensity, conjugate to proteins for targeting, composed of cytotoxic heavy metals, unstable in UV radiation	Used *in vitro* labeling of liver cells, fluorescent assays to detect antigens on cells, used *in vivo* cancer detection and diagnosis	More stable signals than fluorescent molecules, brighter, can bind with proteins

Dendrimers	5–20 nm highly branched macromolecules synthesized through polymerization reaction, growing outward from a central core 5–10, branched structure allows high drug carriage	Cause dose and surface charge dependent hemolysis, cytotoxic *in vitro*, targeted delivery of drugs in aqueous space within liposome or intercalated in to lipid bilayers, used in gene delivery	Show polymerization, terminal groups can be modified for drug targeting, show high drug carriage

Fullerenes	1.5–5000 length and 0.5–20 diameter, very similar to carbon nanotubes an extended *π* conjugated carbon skeletons Vaporization of graphites	Heterofullerenes, 13 C labeled fullerenes, azafullerens	Higher drug delivery for brain tumors

## Abbreviations

BBB: Blood brain barrier
CNS: Central nervous system
BK: Bradykinin
NO: Nitric oxide
sGC: Soluble guanylate cyclase
ABC cassettes: ATP binding cassettes
cRGD: Cyclic arginine-glycine-aspartic acid
DPPC: Dipalmitoylphosphatidylcholine
ELP: Elastin-like polypeptide
NRP: Receptor neuropilin-1
BDNF: Brain derived neurotropic factor
p-gp: p-Glycoprotein
LDL: Low-density lipoprotein
MRI: Enhance magnetic resonance imaging
NPs: Nanoparticles
HAD: HIV-1 associated dementia
NACA: N acetylcysteine amide
PKC: Protein kinase
AD: Alzheimer's disease
HIR: Human insulin receptor
MAb: Monoclonal antibody
PEDF: Pigment epithelium-derived factor
GDEPT: Gene-directed enzyme prodrug therapy
ADEPT: Antibody-directed enzyme prodrug therapy.
